# Signal peptide mimicry primes Sec61 for client-selective inhibition

**DOI:** 10.1038/s41589-023-01326-1

**Published:** 2023-05-11

**Authors:** Shahid Rehan, Dale Tranter, Phillip P. Sharp, Gregory B. Craven, Eric Lowe, Janet L. Anderl, Tony Muchamuel, Vahid Abrishami, Suvi Kuivanen, Nicole A. Wenzell, Andy Jennings, Chakrapani Kalyanaraman, Tomas Strandin, Matti Javanainen, Olli Vapalahti, Matthew P. Jacobson, Dustin McMinn, Christopher J. Kirk, Juha T. Huiskonen, Jack Taunton, Ville O. Paavilainen

**Affiliations:** 1 Institute of Biotechnology, HiLIFE, University of Helsinki, Helsinki, Finland; 2 Department of Cellular and Molecular Pharmacology, University of California, San Francisco, CA USA; 3 Kezar Life Sciences, South San Francisco, CA USA; 4 Institute of Virology, Charité-Universitätsmedizin Berlin, Corporate Member of Freie Universität Berlin and Humboldt-Universität zu Berlin, Berlin, Germany; 5 Department of Pharmaceutical Chemistry, Faculty of Pharmacy, University of California, San Francisco, CA USA; 6 Department of Virology, Faculty of Medicine, University of Helsinki, Helsinki, Finland

**Keywords:** Drug discovery, Structural biology, Protein folding

## Abstract

Preventing the biogenesis of disease-relevant proteins is an attractive therapeutic strategy, but attempts to target essential protein biogenesis factors have been hampered by excessive toxicity. Here we describe KZR-8445, a cyclic depsipeptide that targets the Sec61 translocon and selectively disrupts secretory and membrane protein biogenesis in a signal peptide-dependent manner. KZR-8445 potently inhibits the secretion of pro-inflammatory cytokines in primary immune cells and is highly efficacious in a mouse model of rheumatoid arthritis. A cryogenic electron microscopy structure reveals that KZR-8445 occupies the fully opened Se61 lateral gate and blocks access to the lumenal plug domain. KZR-8445 binding stabilizes the lateral gate helices in a manner that traps select signal peptides in the Sec61 channel and prevents their movement into the lipid bilayer. Our results establish a framework for the structure-guided discovery of novel therapeutics that selectively modulate Sec61-mediated protein biogenesis.

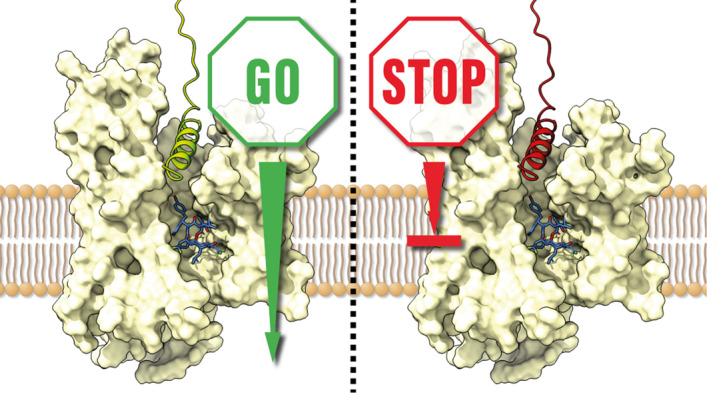

## Abstract (web summary)

A selective inhibitor of Sec61 blocks protein entry into the secretory pathway and has therapeutic efficacy in rheumatoid arthritis. A cryo-EM structure of the inhibited Sec61 provides a model for client-selective protein translocation inhibition.

## Main

Secretory and integral membrane protein biogenesis occurs primarily at the surface of the endoplasmic reticulum (ER). Such proteins, including cytokines and cell-surface receptors, comprise approximately one-third of the eukaryotic proteome^1^ and have critical roles in cancer and inflammatory diseases. Biogenesis of most membrane and secreted proteins requires the Sec61 translocon^2^, an evolutionarily conserved transmembrane channel complex that facilitates translocation and membrane integration of nascent polypeptides. As a key nexus of the secretory pathway, pharmacological modulation of Sec61 could potentially prevent the biogenesis of proteins with critical roles in intercellular communication and disease physiology.

A defining feature of Sec61 clients is the presence of an N-terminal signal peptide or TM helix, which is required for cotranslational ER targeting and insertion^3^. Signal peptides are typically 15–45 amino acids in length. Although they lack a clear consensus motif, signal peptides generally consist of a positively charged N-terminal region, a central hydrophobic region that binds transiently to Sec61, and a C-terminal region containing the signal peptidase cleavage site^4^. A nascent protein’s signal peptide or N-terminal TM segment exits the ribosome and initially engages the cytosolic signal recognition particle (SRP)^5^. This ribosome–nascent chain complex (RNC) is then targeted to the ER membrane and delivered to the Sec61 translocon. RNC docking has been proposed to alter the conformation of Sec61 to prime it for polypeptide insertion^2^. A structure of mammalian Sec61 in complex with an inserted signal peptide^6^ revealed that the hydrophobic region intercalates between Sec61 TM helices (the ‘lateral gate’) and thereby gains access to the lipid bilayer. Hence, a primary mechanism by which signal peptides promote the opening of the Sec61 channel is through dynamic hydrophobic interactions with residues of the lateral gate helices TM2 and TM7. Despite the stringent requirement for recognition by the Sec61 complex, signal peptides are remarkably diverse in sequence and, therefore, may interact with Sec61 lateral gate helices in different ways during the dynamic translocation process.

Consistent with its essential role, complete blockade of Sec61-mediated protein import with small-molecule inhibitors is eventually toxic to mammalian cells^7–11^. A few Sec61 inhibitors, including mycolactone, apratoxin and their derivatives, have been tested in preclinical disease models (primarily cancer). However, these compounds lack Sec61 client selectivity and have generally suffered from a low therapeutic index, precluding further development^12^. By contrast, certain cotransin cyclic heptadepsipeptides potently inhibit Sec61 in a client-selective manner^13,14^. The main determinant of client-selective inhibition appears to be the primary amino acid sequence and corresponding biophysical properties of the N-terminal signal peptide or the TM segment. Previous work demonstrated that cotransin directly targets the Sec61α subunit of the heterotrimeric Sec61 channel^15^, and unbiased mutagenesis screens suggested that cotransins bind Sec61α near the lumenal plug and the lateral gate^16,17^. Remarkably, modifications to the cotransin structure can alter the range of inhibited Sec61 clients^18^, suggesting the possibility of identifying cotransin variants that affect distinct subsets of secretory and membrane proteins. However, the lack of a molecular-level understanding of how cotransins bind Sec61 to achieve substrate-selective effects has prevented the rational design of cotransins with altered pharmacological profiles.

Here we present a cryogenic electron microscopy (cryo-EM) structure of Sec61 bound to a new cotransin variant **1**, hereafter referred to as KZR-8445. KZR-8445 prevents the biogenesis of a subset of Sec61 clients and is efficacious in a mouse model of rheumatoid arthritis. Our cryo-EM structure reveals that KZR-8445 binds to the central region of the Sec61 lateral gate in an open configuration, with direct contact with the lumenal plug. Similar to nascent signal peptides, KZR-8445 disrupts the interhelical lateral gate interactions that stabilize the channel in its closed configuration. Based on our structure, we propose a mechanism in which inhibitor binding to the open lateral gate and closed lumenal plug specifically traps nascent signal peptides in the cytosolic vestibule, thus providing a framework for the design of new cotransins with increased levels of client discrimination.

## Results

### A client-selective Sec61 inhibitor with in vivo efficacy

**Fig. 1:**
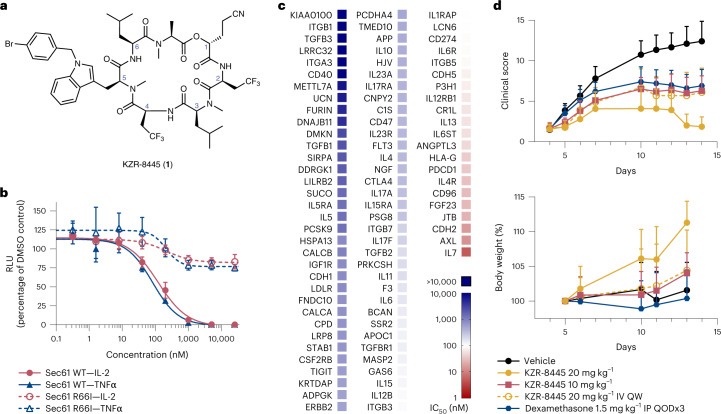
KZR-8445 is a client-selective Sec61 inhibitor and is efficacious in a mouse arthritis model. **a**, Chemical structure of KZR-8445. **b,** Cells stably expressing WT or R66I Sec61α were transfected with dox-inducible Gaussia luciferase (GLuc) reporter constructs fused to the C-terminus of IL-2 or TNF. After treatment with doxycycline and the indicated concentrations of KZR-8445 for 24 h, GLuc activity was quantified. Data are mean values ± s.d. from a single independent experiment. **c**, Cells were transfected with dox-inducible GLuc constructs fused to the indicated human signal peptides (SP-GLuc), treated with doxycycline and increasing concentrations of KZR-8445 for 24 h and assessed for GLuc activity. The heat map depicts IC_50_ values calculated from each SP-GLuc dose–response curve. **d**, Arthritis was induced in BALB/c mice with antibodies specific for type II collagen (mAb) on day 0 and endotoxin on day 3. On day 4, when the disease was present in all animals, mice were randomized and treated for 2 weeks with thrice weekly (QODx3) or weekly (QW) intravenous administration of vehicle or KZR-8445 or intraperitoneal administration of dexamethasone. Clinical scores (0–4 per paw; *n* = 10 per group) and body weights were followed until day 14. Data are presented as mean values ± s.d. mAb, monoclonal antibody. Source data

**Extended Data Fig. 1:**
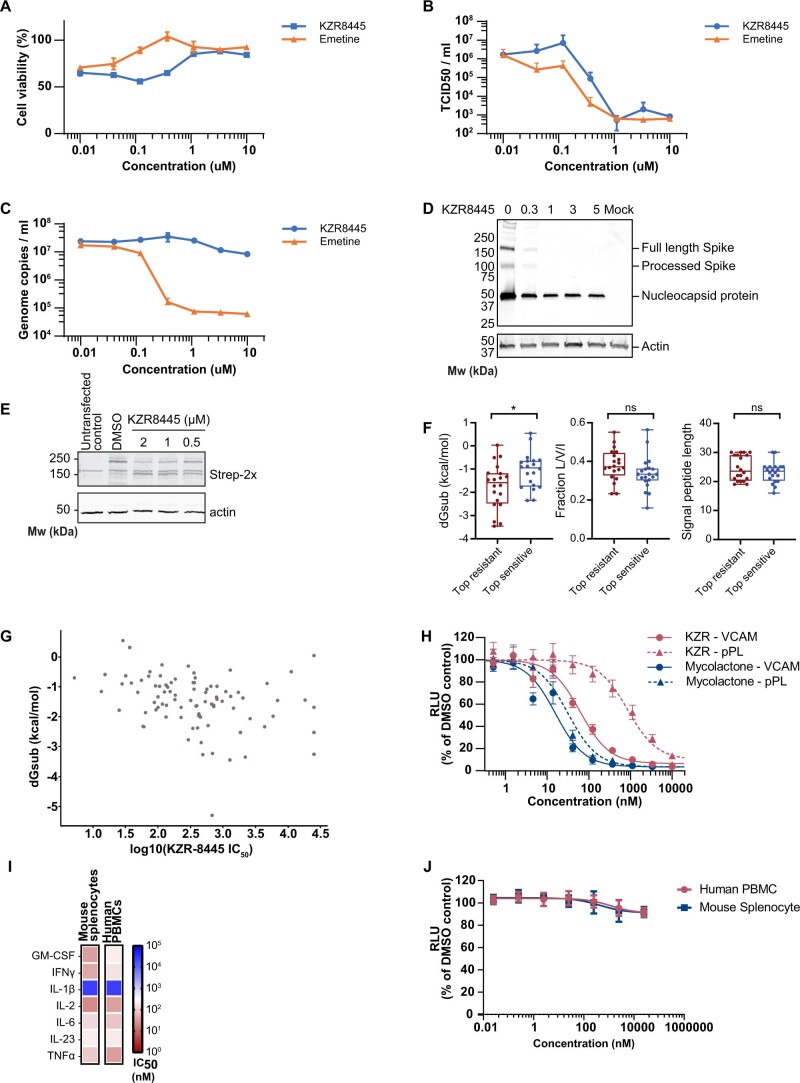
Cotransin KZR-8445 inhibits SARS-CoV-2 viral replication and biogenesis of the Spike protein. **(a–c)** Effect of KZR-8445 and emetine on cell viability **(a)**, infectious virus particle production **(b)**, and viral genome copy number **(c)** in Vero E6 cells infected with SARS-CoV-2 for 48 h. Data are shown as mean values ± SD (n = 3 replicates) from a single experiment. **(d)** Effect of KZR-8445 on Spike protein biogenesis in Vero E6 cells infected with SARS-CoV-2 for 48 h, assessed by immunoblotting. ‘Mock’: mock-infected cells. **(e)** Effect of KZR-8445 on SARS-Cov-2 Spike protein (2xStrep-tagged) transiently overexpressed in HEK293 cells. Data are representative of two independent experiments. **(f)** Comparison of signal peptide (SP) features from the top-20 most resistant versus top-20 most sensitive SP-GLuc IC50 values (related to Fig. 1c). dGsub was calculated for each SP using https://dgpred.cbr.su.se/index.php?p=TMpred (subsequence allowed; no length correction). P-value for dGsub (*) is 0.0282 (Wilcoxon rank-sum one-sided test; ns, not significant). **(g)** Scatterplot of SP-GLuc log(IC_50_) values (nM) (Fig. 1c) versus calculated dGsub. (**h**) Effect of KZR-8445 and mycolactone A/B on the secretion of eGluc2 fused to the signal peptide of human VCAM or bovine preprolactin (pPL) in transiently transfected HEK293T cells. Secreted luciferase data (RLU, relative light units) are shown as mean ± SD (n = 3 replicates), representative of two independent experiments. **(i)** Effect of KZR-8445 on cytokines secreted from human peripheral blood mononuclear cells (PBMCs) or mouse splenocytes stimulated with lipopolysaccharide or antibodies against CD3 and CD28 (see Methods). After 24 h, supernatants were collected for cytokine analysis by MSD U-PLEX electrochemiluminescent immunoassay and IC50 values calculated from dose-response curves. **(j)** Effect of KZR-8445 on cell viability of unstimulated human PBMCs and mouse splenocytes (CTG assay, 24 h treatment). Data are shown as mean values ± SD from 4 (PBMCs) or 2 (splenocytes) independent experiments. Source data

The previously identified cotransins, CT8 and PS3061, potently block the secretion of pro-inflammatory cytokines^18^ and inhibit viral replication^19,20^, including severe acute respiratory syndrome coronavirus 2 (SARS-CoV-2)^21^. To test whether cotransins can serve as disease-modifying agents in vivo, we designed KZR-8445 (Fig. 1a), a fluorinated variant of PS3061 with improved physicochemical properties and pharmacokinetics. Similar to PS3061, KZR-8445 blocked CoV-2 replication and virus-induced cytotoxicity in Vero cells, an effect that is likely mediated by inhibition of spike protein biogenesis (Extended Data Fig. 1a–e). KZR-8445 was also recently shown to overcome dexamethasone resistance in T-cell acute lymphoblastic leukemia cells in vitro^22^.

To investigate the effects of KZR-8445 on secretory and membrane protein biogenesis and to establish Sec61α as the relevant target, we quantified the secretion of a doxycycline-inducible Gaussia luciferase (GLuc) reporter fused to the C-terminus of two full-length pro-inflammatory cytokines, interleukin-2 (IL-2) (which contains a cleavable signal peptide) and tumor necrosis factor-alpha (TNFα) (which contains an N-terminal transmembrane anchor). We performed the GLuc secretion assays in cells stably expressing exogenous wild type (WT) Sec61α or a plug domain mutant (R66I) that was previously shown to confer resistance to CT8 and a cotransin natural product^16,17^ (Fig. 1b). KZR-8445 potently blocked secretion of both GLuc reporter constructs (IC_50_ ~ 100 nM), and this effect was reduced in cells expressing R66I Sec61α. Hence, KZR-8445 acts directly on Sec61α to block protein secretion. KZR-8445 is structurally related to CT8, which discriminates among Sec61 clients based largely on their N-terminal signal peptide^18^. To test whether KZR-8445 exhibits signal peptide-dependent selectivity, we screened a panel of 89 signal peptide-GLuc reporter constructs derived from diverse disease-relevant Sec61 clients. Results from this screen revealed a striking variation of signal peptide sensitivity to KZR-8445, with IC_50_s ranging from 5 nM to >25 µM (median = 366 nM), depending on the signal peptide (Fig. 1c). Although the biophysical basis of signal peptide sensitivity requires further investigation, we observed a significant correlation between KZR-8445 sensitivity and signal peptide hydrophobicity (Spearman’s rank, *ρ* = −0.34, *P* = 0.001; Extended Data Fig. 1f,g), which we calculated using the ‘biological hydrophobicity’ scale described in refs. ^23,24^. We also directly compared KZR-8445 to mycolactone A/B, a structurally distinct and relatively client-nonselective Sec61 inhibitor^8,25^, for their ability to discriminate between vascular cell adhesion molecule (VCAM) and preprolactin (pPL) signal peptides. Although KZR-8445 inhibited VCAM-GLuc 20-fold more potently than pPL-GLuc, mycolactone A/B was equipotent against both reporters (Extended Data Fig. 1h). These results further highlight a unique signal peptide inhibition profile and suggest that KZR-8445 might exert a distinct mechanism of Sec61 modulation as compared with mycolactone A/B. Finally, we tested the effects of KZR-8445 on the production of endogenous pro-inflammatory cytokines by activated human peripheral blood mononuclear cells (PBMCs) and mouse splenocytes (Extended Data Fig. 1i,j). KZR-8445 blocked the secretion of six of seven tested cytokines with submicromolar IC_50_s, while having minimal effects on cell viability at concentrations up to 20 µM. Notably, secretion of IL-1β, which is not Sec61-dependent, was unaffected by KZR-8445.

Although the nonselective Sec61 inhibitor mycolactone A/B showed modest efficacy in a mouse skin inflammation model when tested at its maximum tolerated dose^26^, client-selective Sec61 modulators have not been evaluated in animal models of chronic inflammatory disease to the best of our knowledge. To determine if the anticytokine secretion effects observed in vitro would translate to an anti-inflammatory effect in vivo, we tested KZR-8445 in a collagen antibody-induced mouse model of rheumatoid arthritis. When administered thrice weekly after the onset of the disease, KZR-8445 blocked disease progression in a dose-dependent manner (10 mg kg^−^^1^, *P* = 0.01; 20 mg kg^−^^1^, *P* < 0.0001, one-way analysis of variance (ANOVA) versus vehicle). At the highest dose, KZR-8445 produced a stronger anti-inflammatory effect than the corticosteroid dexamethasone. Weekly administration of KZR-8445 was also effective (20 mg kg^−^^1^, *P* = 0.007). Finally, none of the KZR-8445 treatment groups experienced significant toxicity as measured by body weight changes (Fig. 1d). Collectively, our data show that KZR-8445 is a potent and substrate-selective inhibitor of Sec61. Unlike other previously described Sec61 inhibitors, KZR-8445 is efficacious and well-tolerated in an animal model of rheumatoid arthritis.

### Cryo-EM analysis of Sec61 bound to KZR-8445

**Extended Data Fig. 2:**
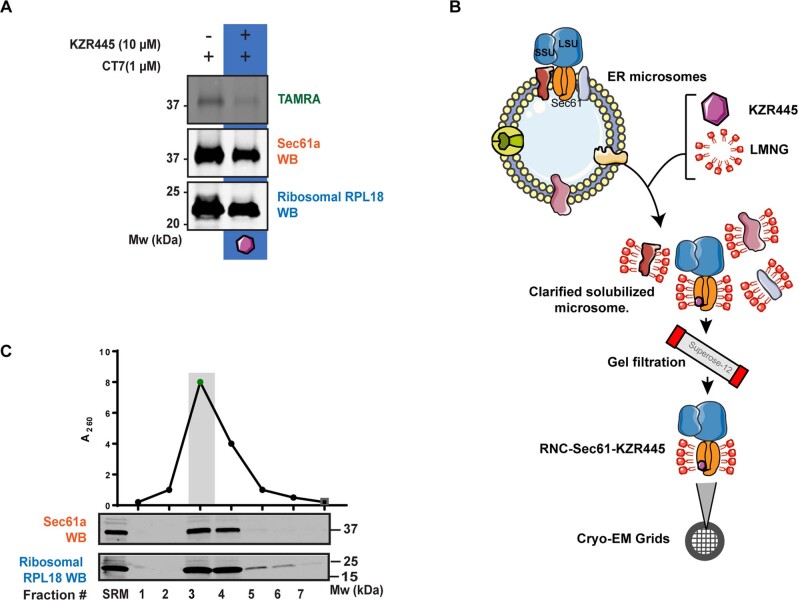
Isolation and biochemical characterization of the inhibitor-bound ribosome-Sec61 (RNC) complexes. **(a)** Photocrosslinking experiment showing competitive binding of KZR-8445 with photo-cotransin (CT7) in the final purified sample. Highlighted sample (gray) was used for cryo-EM analysis. Gels are representative examples of n = 3 independent experiments. **(b)** Flowchart of purification of KZR-8445-bound RNC–Sec61 complex. Isolated sheep pancreatic rough ER microsomes were supplemented with 1 µM inhibitor and incubated on ice for 30 min followed by solubilization with 1% LMNG for 1 h. Solubilized material was clarified by centrifugation followed by gel filtration. **(c)** Gel filtration profile of clarified detergent-solubilized sample fractions with measured absorbance at A_260_. Western blot analysis demonstrates presence of Sec61 and ribosomes in the peak fractions confirming integrity of the purified complex. Gels are representative examples of n = 3 independent experiments. Source data

To understand the molecular basis of Sec61 inhibition by KZR-8445, we pursued structural investigations using single-particle cryo-EM. Our initial attempts to isolate cotransin-bound ribosome–Sec61 complexes with the commonly used detergent digitonin were not successful. Several other detergents were screened for retention of native-like cotransin binding by subjecting solubilized Sec61 to a cotransin photo-affinity labeling assay^15^ (Extended Data Fig. 2a). This screen identified lauryl maltoside neopentyl glycol (LMNG), a lipid-mimetic detergent that permits maximal photo-cotransin binding, as indicated by specific crosslinking to the Sec61α subunit in LMNG-solubilized ER membranes. Notably, photo-cotransin crosslinking was efficiently competed not only by excess KZR-8445 but also by a structurally unrelated Sec61 inhibitor apratoxin A^11^, suggesting that LMNG solubilization may allow preparation of Sec61 complexes bound to other inhibitors.

**Extended Data Fig. 3:**
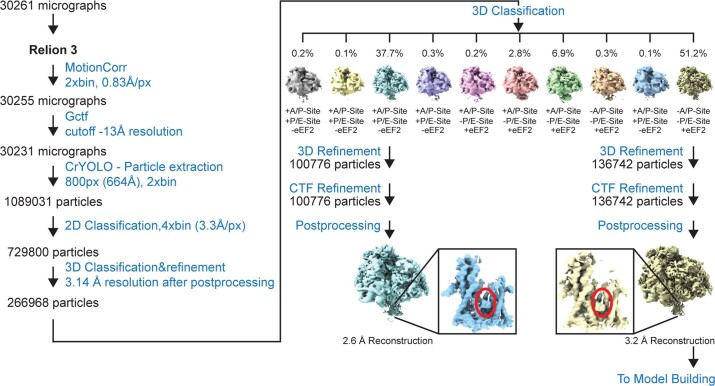
Cryo-EM data-processing workflow for ribosome-Sec61 translocon complexes.

**Extended Data Fig. 4:**
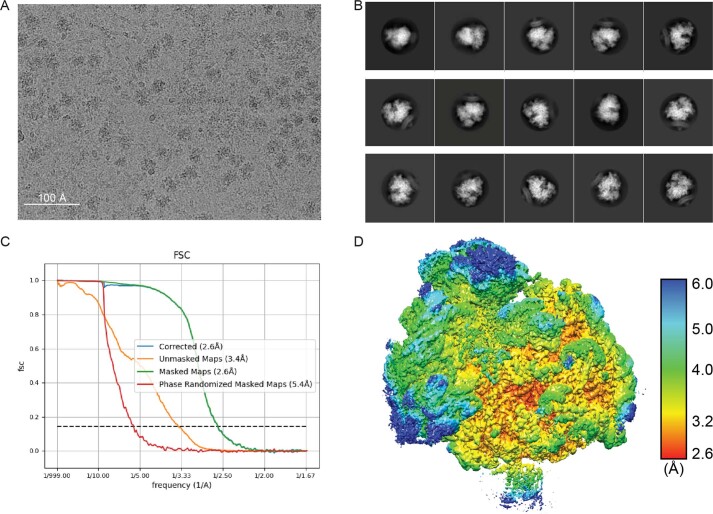
Cryo-EM characterization of the ribosome/Sec61/KZR-8445 complex. **(a)** A representative micrograph of ribosome-Sec61 complex on holey carbon grids coated. **(b)** Reference free 2D class averages showing different views of the ribosomes/Sec61 complex. **(c)** Fourier shell correlation (FSC) curves of the final reconstruction map. **(d)** Local resolution of the ribosome/Sec61 complex. Source data

Having established solubilization conditions that preserve native-like KZR-8445 binding to Sec61, we proceeded with isolation of the ribosome–Sec61–KZR-8445 complex for single-particle cryo-EM structure determination. We solubilized sheep ER microsomes that were pretreated with 10 µM KZR-8445, isolated the ER-bound 80S polysomes, converted them to 80S monosomes by RNAse treatment and subjected them to gravity-flow size exclusion chromatography (Extended Data Fig. 2b). The fractions containing intact ribosome–Sec61 complexes (Extended Data Fig. 2c) were deposited on holey cryo-EM grids with thin carbon support. We collected a dataset consisting of 30,266 micrographs (Extended Data Table 1) and carried out single-particle structure determination (Extended Data Fig. 3). This resulted in a consensus reconstruction with an overall resolution of 3.2 Å and local resolution in the Sec61 region of 2.6–7.0 Å, with poorest density in the N-terminal half of the channel (Extended Data Fig. 4).

**Fig. 2:**
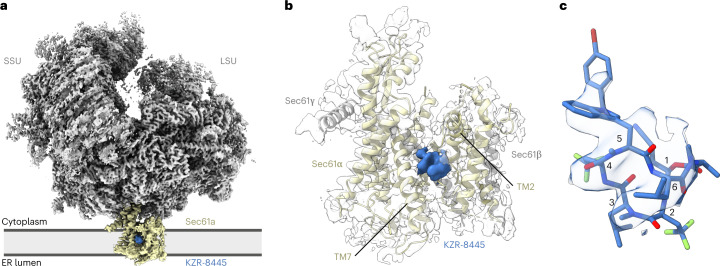
Structure of the mammalian Sec61 translocon with KZR-8445, a substrate-selective translocation inhibitor. **a**, Cryo-EM map of the mammalian ribosome-bound heterotrimeric Sec61 translocon in complex with the cotransin analog KZR-8445. The map was low-pass filtered to 4 Å with density features corresponding to the ribosome (LSU and SSU), Sec61 and KZR-8445. **b**, Additional density assigned to KZR-8445 is located between TM2, TM3, TM7 and lumenal plug helices. KZR-8445 is bound to the center of the lateral gate, which adopts an open conformation. **c**, Fit of KZR-8445 to the observed density. LSU, ribosomal large subunit; SSU, ribosomal small subunit.

**Extended Data Fig. 5:**
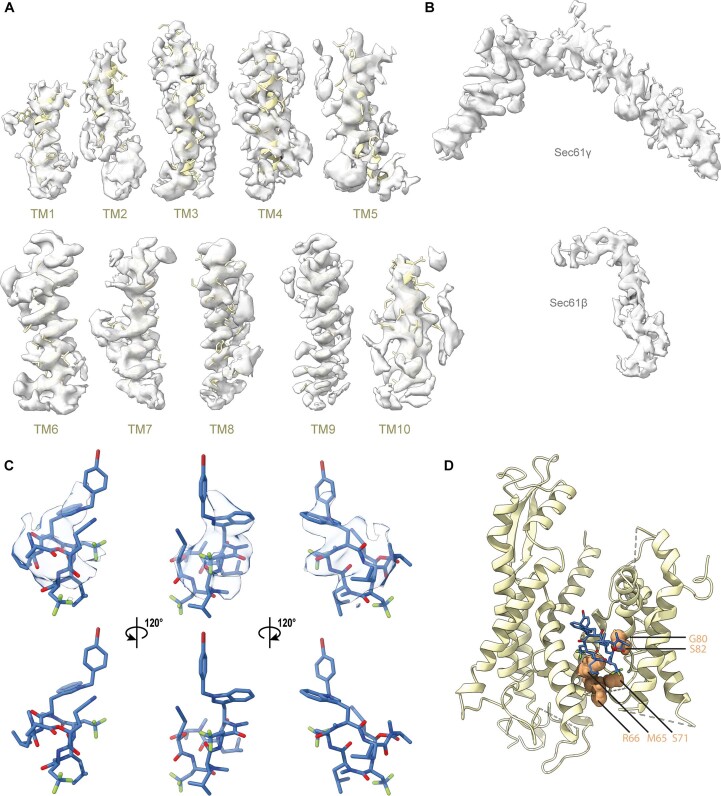
Structure of the mammalian Sec61 translocon with a substrate-selective translocation inhibitor cotransin. **(a)** Density of the fitted map within 3 Å of each transmembrane helix of Sec61α shown at 1.5 σ. **(b)** Density of the fitted map within 3 Å of Sec61γ (top) or Sec61β (bottom), shown at 1.5 σ. **(c)** Different views of density map about KZR-8445. **(d)** Locations of mutations conferring resistance to various Sec61 inhibitors.

Consistent with previous structural work^6,27,28^, the obtained cryo-EM map revealed a well-defined ribosome density, with additional density representing the trimeric Sec61 translocon proximal to the ribosome exit tunnel (Fig. 2a and Extended Data Fig. 5a,b). The quality of the cryo-EM density allowed unambiguous modeling throughout most of the C-terminal half of Sec61α, while the weaker density representing the N-terminal half likely reflects the greater conformational flexibility of this part of the channel. In addition to the density corresponding to Sec61 subunits, we observed a prominent density in the center of the channel, near the lumenal plug and connected to Sec61 TM7 (Fig. 2b and Extended Data Fig. 5c). The additional density is proximal to Sec61α residues that, when mutated, were previously found to confer resistance to various cotransin analogs^16,17^ (Extended Data Fig. 5d). The overall shape and size of the presumed KZR-8445 density fits the expected conformation of KZR-8445 as determined by molecular modeling (Fig. 2c and Extended Data Fig. 5c). Furthermore, we observe the same density in both translating and nontranslating ribosomes as assessed by the presence of A/P and P/E-site tRNAs, suggesting that the additional density does not belong to a nascent polypeptide. We conclude that the extra density in the Sec61 channel likely represents bound KZR-8445.

### KZR-8445 interacts with the lateral gate of Sec61

**Fig. 3:**
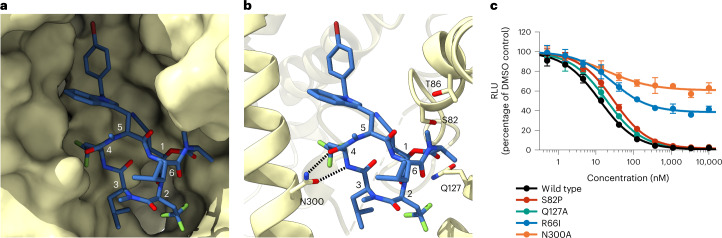
Detailed view of the KZR-8445 binding site. **a,** Solvent-excluded surface view of the open Sec61α lateral gate bound to KZR-8445. **b**, Polar residues of the Sec61α cavity proximal to KZR-8445. **c**, KZR-8445 sensitivity of a VCAM-SP Gluc reporter construct in cells expressing the indicated Sec61α mutant. Data are mean values ± s.d. from *n* = 2 independent experiments. Source data

To create a model of Sec61 bound to KZR-8445, we used the closed model of mammalian Sec61 (ref. ^27^) as a starting template. Real-space refinement and manual modeling revealed that the KZR-8445 macrocycle is inserted into a fully opened lateral gate (Fig. 3a). We note that the conformation of the Sec61 lateral gate in our modeled structure is generally similar to that reported for the yeast post-translational translocon^29,30^, as well as the structure of mycolactone A/B bound to a distinct site near the cytosolic vestibule of mammalian Sec61 (ref. ^31^). Therefore, the Sec61 conformation in our structure represents an energetically favorable state, which may exist during the dynamic protein translocation process.

We initially modeled KZR-8445 into the central cavity of Sec61 by identifying the likely low-energy conformations of the macrocycle^32,33^. This analysis revealed two distinct backbone conformations of KZR-8445 with the main difference being a *cis* or *trans N*-methyl amide bond linking R-2 and R-3 residues. We fitted both models into the cryo-EM density and obtained a better match with the *cis* conformation of KZR-8445, which is similar to the conformation of the related cotransin natural product, HUN-7293, as determined by nuclear magnetic resonance and x-ray crystallography^34^. We, therefore, used this conformation to model KZR-8445 into the cryo-EM density. Initial positioning of KZR-8445 within Sec61 was facilitated by assigning a prominent feature of the density to the R-5 bromobenzyl-tryptophan side chain (Fig. 2c and Extended Data Fig. 5c). The lumenal plug of Sec61 is visible in the cryo-EM density, indicating that it is ordered when bound to KZR-8445. We note that the resolution of the KZR-8445 density itself is limited to 4–5 Å, and the smaller KZR-8445 side chains are, therefore, unresolved in our map.

**Extended Data Fig. 6:**
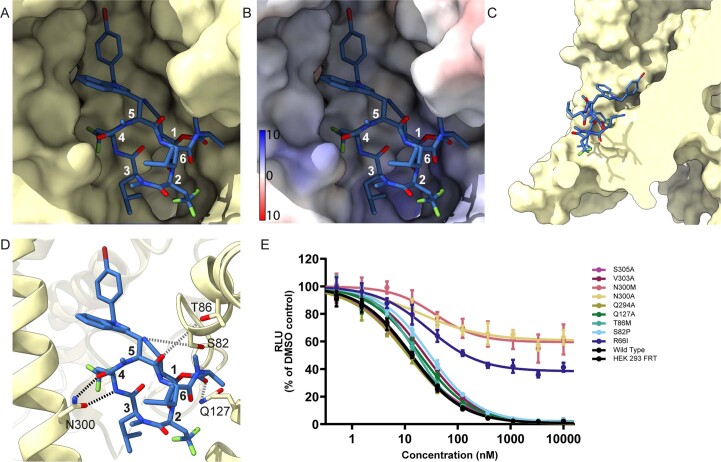
KZR-8445 interfaces Sec61 and the lipid membrane in a multifaceted manner. **(a)** Solvent excluded surface view of the KZR-8445 binding site within Sec61 viewed from the direction of the open lateral gate. **(b)** Same view, but surface colored based on the Coulombic potential surface. **(c)** Side view of the solvent excluded surface of the KZR-8445 binding site with intervening surfaces rendered transparent. Part of KZR-8445 is exposed to the lipid bilayer through the open lateral gate. **(d)** Polar residues of the Sec61α cavity proximal to KZR-8445**. (e)** KZR-8445 sensitivity of luciferase reporter constructs expressed in the background of Sec61 mutant cell lines in engineered human HEK293 cells. Data are mean values ± SD and are representative of n = 2 independent experiments. Source data

While our manuscript was under review, a cryo-EM structure of a cotransin analog bound to a humanized mutant of the yeast post-translational translocon was reported^35^. This higher resolution structure prompted us to compare our initial model with an alternative in which the Sec61 TM7 register was shifted by one amino acid. To accommodate this shift, we rotated KZR-8445 slightly with respect to the channel axis. Our second model provided a better overall fit of TM7 and part of the TM6-7 loop into the experimental density. In addition, the second model revealed two potential hydrogen bonds between the side chain carboxamide of TM7 residue N300 and the R-4 backbone amide and carbonyl of KZR-8445 (Fig. 3b). We, therefore, used the second model in all subsequent analyses. In this arrangement, KZR-8445 is bounded by the lateral gate and lumenal plug helices of Sec61 and is enclosed by the hydrophobic interior of the lipid bilayer, thereby occluding the channel pore and restricting access to the membrane (Fig. 3a and Extended Data Fig. 6a–c).

Additional Sec61α residues that could potentially form polar interactions with KZR-8445 include S82, T86 and Q127 (Extended Data Fig. 6d). We note that T86, Q127 and N300 have been suggested to form a network of intramolecular hydrogen bonds that maintain the closed state of the channel^36^. Conceivably, competition with these interactions could contribute to the mechanism by which KZR-8445 opens the Sec61 lateral gate. Consistent with a critical role for N300, ectopic expression of N300A Sec61α in HEK293 cells conferred resistance to KZR-8445 in the secreted GLuc assay (Fig. 3c and Extended Data Fig. 6e). Although R66 does not form polar contacts with KZR-8445 based on our model, mutation of this residue (R66I) also conferred resistance, possibly due to an altered conformation of the plug helix. By contrast, mutation of other proximal polar residues had little or no effect in this assay. We conclude that Sec61α N300 is essential for KZR-8445-mediated inhibition of protein secretion. Competition with intramolecular interactions between N300, T86 and Q127, which stabilize the lateral gate in its closed configuration, may allow KZR-8445 to open the lateral gate in the absence of a signal peptide.

**Extended Data Fig. 7:**
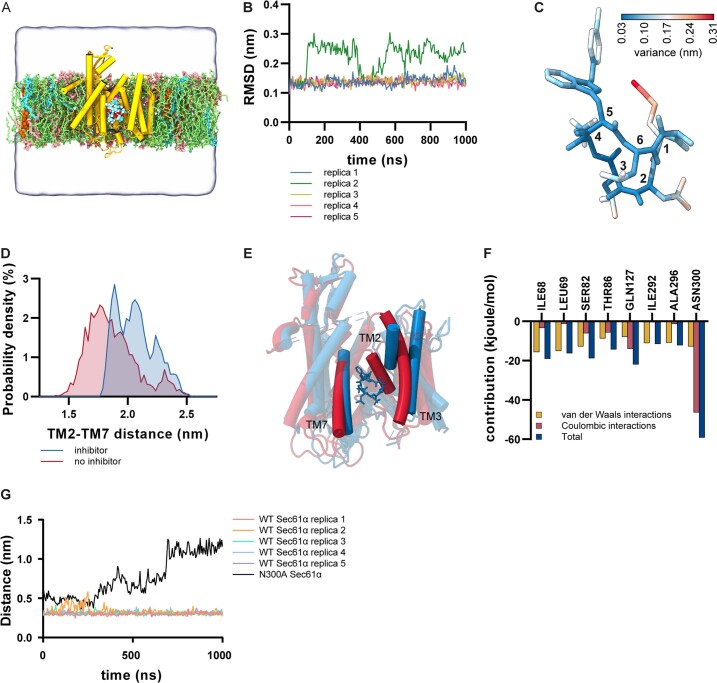
Molecular dynamics (MD) simulations of Sec61 bound to KZR-8445 in an intact membrane environment. **(a)** Simulation system. Sec61 is shown in yellow and KZR-8445 based on chemical identity. Zwitterionic lipids (POPC, POPE, PSM) are shown in green, POPS in cyan, POPI in pink, and cholesterol in orange. Water is rendered as a transparent surface to highlight the simulation box dimensions, and ions are omitted for clarity. **(b)** Root mean squared deviation (RMSD) values for KZR-8445 as a function of simulation time, relative to its starting conformation. In one replica, the backbone around the nitrile group switches between two conformations, leading to large jumps in RMSD values. **(c)** Root mean squared fluctuation (RMSF) values mapped onto the KZR-8445 structure. The ring structure and the indole group reside stably in one conformation, whereas the trifluoromethyl groups are somewhat more mobile. The large RMSF values of the nitrile group mainly derive from a conformational change in the backbone observed in one simulation replica, leading to a different orientation of the nitrile group. The bromophenyl group rotates freely, leading to larger RMSF values. **(d)** The probability density of the TM2–TM7 distance that describes the degree of openness of the lateral gate. **(e)** Final structures (after 1 µs of MD simulation) of Sec61 with KZR-8445 present (blue) or without KZR-8445 (red). Both simulations were initiated from an open conformation. **(f)** Sec61 residues interacting with KZR-8445 with either van der Waals or Coulombic energy greater than 2 kcal/mol (8.4 kJ/mol). **(g)** Shortest distance between Sec61 N300 (or A300 in the mutant) and the peptide bond nitrogen between R-3 and R-4 in KZR-8445 as a function of simulation time. KZR-8445 remains stably bound in WT Sec61, but detaches from its binding site during the 1 µs MD simulation in the N300A mutant. Source data

We used atomistic molecular dynamics (MD) simulations to qualitatively assess the proposed KZR-8445 binding mode. Our simulations included the KZR-8445-bound Sec61 complex embedded in a lipid bilayer that closely mimics the known ER lipid composition^37^, surrounded by explicit solvent (Extended Data Fig. 7a). Repeated microsecond-long MD simulations suggest that the modeled pose of KZR-8445 is stable (Extended Data Fig. 7b), with the R-1 nitrile side chain having the largest conformational variability (Extended Data Fig. 7c). The simulations also suggest that KZR-8445 maintains the lateral gate in an open conformation because its removal resulted in partial closure of the lateral gate (Extended Data Fig. 7d,e). Hydrogen bonds between the carboxamide of N300 and the R-4 backbone carbonyl and amide groups of KZR-8445 (Fig. 3b) were present during 91% of the accumulated 4 µs simulation time, with other residues primarily contributing van der Waals interactions (Extended Data Fig. 7d). The significance of the hydrogen bonding interactions, confirmed by mutagenesis (Fig. 3c), is further suggested by a simulation with the N300A mutant, which showed a complete loss of binding (Extended Data Fig. 7g). Collectively, the structural observations, location of cotransin resistance mutations and MD simulations support the proposed binding mode of KZR-8445 within the lumenal cavity of Sec61α, as well as a critical role for N300 in KZR-8445 binding and inhibition.

### Structural insights lead to improved client selectivity

**Fig. 4:**
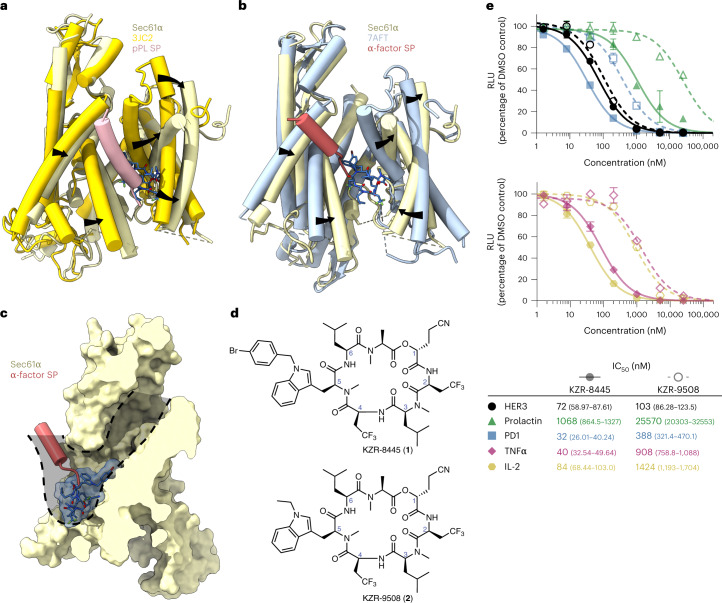
Structural insights lead to improved signal peptide selectivity. **a**, Superimposition of Sec61–KZR-8445 complex with preprolactin signal peptide (salmon) engaged with Sec61 (PDB: 3JC2, bright yellow). **b**, Superimposition of Sec61–KZR-8445 complex with yeast α-factor signal peptide (red) engaged with Sec61 (PDB: 7AFT, blue). **c**, Sec61/KZR-8445 superimposed with a yeast α-factor signal peptide showing the solvent-excluded surface of the translocon. Indicated in gray is a putative route traversed by nascent signal peptides to reach the binding site occupied by the yeast α-factor signal peptide. **d**, Structures of KZR-8445 and KZR-9508, a cotransin with a truncated R-5 side chain. **e**, Cells were stably transfected with dox-inducible Gaussia luciferase (GLuc) reporter constructs fused to the C-terminus of the indicated signal peptides (top), or full-length IL-2 or TNFα (bottom). Following treatment with doxycycline and the indicated concentrations of KZR-8445 for 24 h, GLuc activity was quantified. Data are mean values ± s.d. from a single experiment. Source data

Previous evidence suggested that cotransin-family Sec61 inhibitors compete with nascent signal peptides for opening the lateral gate and inserting into the lipid bilayer. In particular, signal peptide mutants with increased hydrophobicity or helical propensity were better able to compete, as revealed by a right shift in the cotransin dose–response curve^16,38^. To gain structural insight into the competitive relationship between nascent signal peptides and cotransins, we superimposed our structure of KZR-8445/Sec61 with two other Sec61 structures, each of which captures a signal peptide inserted between the lateral gate helices in a distinct manner. Comparison of our structure with the prolactin signal peptide bound to mammalian Sec61 (ref. ^6^) reveals a major outward shift of the lateral gate helices TM2, TM3 and TM7 in the KZR-8445 structure (Fig. 4a). TM2 packs against the hydrophobic signal peptide, which adopts an α-helical conformation and is partially exposed to the lipid bilayer. Strikingly, Sec61 TM2 and the prolactin signal peptide overlap substantially with the KZR-8445 binding site. This analysis indicates that KZR-8445 and the prolactin signal peptide directly compete for binding to a functionally critical site within the Sec61 lateral gate. Compared with most signal peptides tested in the SP-GLuc secretion assay, prolactin is relatively resistant, such that higher concentrations of KZR-8445 are required to inhibit its secretion (IC_50_ ~ 1,000 nM; Extended Data Fig. 1i). Hence, a likely contributing feature to Sec61 client selectivity is the relative affinity of KZR-8445 and the nascent signal peptide for overlapping binding sites within the Sec61 lateral gate.

A cryo-EM reconstruction of yeast Sec61 (post-translational translocon with Sec62/Sec63/Sec71/Sec72) bound to the yeast mating type pheromone α-factor^39^ depicts a related but distinct mode of signal peptide binding to the lateral gate (Fig. 4b). In contrast to the prolactin signal peptide, which tightly intercalates between TM2 and TM7 and occupies the same region as KZR-8445, the α-factor signal peptide forms a shorter helical segment and docks near the cytosolic ends of TM7 and TM8, which only partially overlaps with the KZR-8445 binding site. Nevertheless, the bulky bromobenzyl-Trp R-5 side chain in our cryo-EM model of KZR-8445 projects into the Sec61 cytosolic vestibule and would likely block many signal peptides from accessing this site (Fig. 4c). This observation suggested the possibility of further enhancing signal peptide selectivity by reducing the size of the R-5 side chain, as exemplified by **2** (hereafter referred to as KZR-9508; Fig. 4d). We hypothesized that by removing the steric block afforded by the bromobenzyl-Trp side chain, signal peptides entering the cytosolic vestibule would have greater access to the lateral gate. Moreover, the smaller ethyl-Trp side chain in KZR-9508 could potentially impart decreased intrinsic binding affinity for Sec61, facilitating competitive displacement by signal peptides that are otherwise blocked by KZR-8445. We directly compared KZR-8445 and KZR-9508 against a panel of five Sec61 client-GLuc reporters and found that KZR-9508 exhibited greater selectivity (Fig. 4e). Although KZR-9508 was 10–20-fold less potent than KZR-8445 against four of five GLuc reporters, it was nearly equipotent (IC_50_ ~ 100 nM) against the HER3 GLuc reporter. These data suggest that most KZR-8445-sensitive signal peptides, regardless of their precise Sec61 binding mode, are better able to displace KZR-9508. By contrast, certain signal peptides (including HER3) fail to compete with KZR-9508 for binding to the lateral gate (see Discussion).

## Discussion

Many secreted and integral membrane proteins have causal roles in human disease. Preventing their biogenesis by blocking Sec61-dependent entry into the secretory pathway could have profound therapeutic implications. Substrate-nonselective Sec61 inhibitors, including mycolactone and apratoxin, have been described^12^. However, because Sec61-mediated protein import is an essential process in healthy cells, these inhibitors are probably too toxic to be developed as therapeutics^40^. By contrast, substrate-selective Sec61 inhibitors, including cotransin-related cyclic heptadepsipeptides and CADA-related cyclotriazadisulfonamides^13,14,41^ inhibit the biogenesis of a subset of secreted and membrane proteins. How substrate-selective Sec61 inhibitors engage the dynamic translocation channel and discriminate among Sec61 clients in a signal peptide-dependent manner has remained a mystery. By determining the cryo-EM structure of Sec61 bound to the cotransin-related inhibitor KZR-8445, we provide new insights into how substrate-selective Sec61 inhibition can be achieved, as compared to relatively nonselective inhibitors such as mycolactone.

**Fig. 5:**
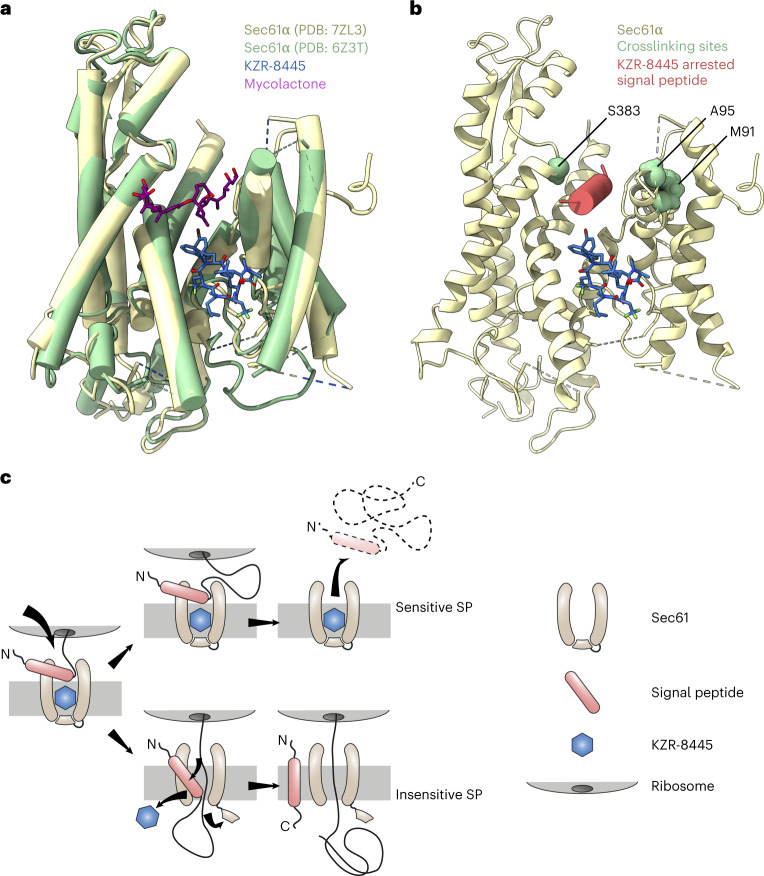
Proposed model for substrate-selective Sec61 inhibition. **a**, Comparison of KZR-8445 (blue) bound to Sec61α (PDB: 7ZL3) and mycolactone (purple) bound to Sec61α (green, PDB: 6Z3T). **b**, KZR-8445-bound Sec61 (wheat) with residues previously shown to crosslink CT8-arrested TNFα highlighted in green. Placement of a putative KZR-8445-arrested signal peptide (red) was guided by the proximity of crosslinked residues. The bulky R-5 group of KZR-8445 projects toward the arrested signal peptide. **c**, Substrate-selective inhibitors, such as KZR-8445, arrest specific signal peptides in a nonproductive conformation in the cytosolic vestibule and in proximity to the KZR-8445 R-5 group, which is important for determining the range of inhibited Sec61 clients. Sensitive signal peptides are unable to progress in the insertion pathway and are displaced into the cytosol. Drug-resistant signal peptides are able to progress further along the insertion pathway. Intercalation between Sec61 lateral gate helices likely leads to inhibitor dissociation and allows translocation of the nascent polypeptide into the ER lumen.

When bound to KZR-8445, the Sec61 lateral gate adopts an open conformation, reminiscent of the open lateral gate observed in the yeast post-translational Sec61 translocon^29,30^. KZR-8445 binds within the central pore of Sec61 at its lumenal end, a site that maps to previously identified cotransin resistance mutations^16,17^. In addition to contacting the lateral gate where signal peptides ultimately insert into the lipid bilayer, KZR-8445 directly contacts the Sec61 plug helix and thereby prevents the Sec61 channel from opening toward the ER lumen. In contrast to KZR-8445, the substrate-nonselective inhibitor mycolactone has been proposed to bind within the cytosolic vestibule based on two independent cryo-EM reconstructions^31,35^ (Fig. 5a), although the precise mycolactone binding site and orientation are different in the two studies. We speculate that because most signal peptides necessarily traverse the cytosolic vestibule before inserting into the lateral gate, steric blockade of the vestibule by inhibitors such as mycolactone would prevent translocation of a broad range of Sec61 client proteins. Mycolactone also binds to Sec61 with an open lateral gate and was recently found to increase calcium leakage from the ER^42^. Whether KZR-8445 and other cotransins affect Sec61-dependent calcium leak is an intriguing question for future studies.

Our structure provides clues into how KZR-8445 achieves substrate-selective Sec61 inhibition and how selectivity can be further enhanced. Superimposition of structures containing a nascent signal peptide bound to Sec61 (refs. ^6,39^) reveals that certain signal peptides (for example, α-factor) dock adjacent to the KZR-8445 binding site, whereas others (for example, prolactin) dock to a lateral gate site that overlaps extensively with KZR-8445. We speculate that in the presence of KZR-8445, a subset of signal peptides stably dock to the cytosolic vestibule (Fig. 5a,b). Our previous work with the cotransin analog CT8 suggests that the N-terminal signal anchor of TNFα falls into this category^16^. The open conformation of Sec61 observed in our structure appears to be compatible with an α-helical signal peptide (or signal anchor) nestled between KZR-8445 and the lateral gate residues S383, A95 and M91 (Fig. 5b). Consistent with this model, these residues were previously shown to reside near the TNFα signal anchor in the context of a pre-insertion complex stabilized by CT8 (ref. ^16^).

Our finding that KZR-9508 is more selective than KZR-8445 suggests that signal peptide selectivity can be tuned by altering the structure of the R-5 side chain. We propose a model for substrate-selective inhibition in which sensitive signal peptides initially engage Sec61 at the cytosolic tip of the lateral gate (Fig. 5c), where they are stabilized in a nonproductive configuration, possibly via direct interactions with a composite surface defined by Sec61 and R-5 of the bound cotransin. This mechanism is reminiscent of nascent chain-selective ribosome inhibitors, which bind to a composite surface defined by the ribosome exit tunnel and specific nascent polypeptide sequences that traverse the exit tunnel^43–45^. Cotransin-resistant signal peptides, on the other hand, can apparently displace the bound inhibitor, intercalate between the lateral gate helices, insert into the lipid bilayer and ultimately promote the opening of the lumenal plug domain.

Secretory proteins, such as TNFα and IL-6, act as inflammatory mediators that promote joint damage during the progression of arthritis^46^. We demonstrated that KZR-8445 inhibits the stimulated secretion of IL-2, TNFα and GM-CSF in primary mouse splenocytes and human PBMCs. We tested KZR-8445 in a collagen antibody-induced mouse model of rheumatoid arthritis^47^, where it ameliorated clinical arthritis symptoms, likely resulting from the selective blockade of several pro-inflammatory secretory proteins. Unlike previously described Sec61 inhibitors that have been tested in animals, KZR-8445 was well-tolerated as evidenced by weight gain with continued dosing, relative to vehicle-treated mice (Fig. 1d). Future structural studies of Sec61 bound to defined signal peptides and client-selective cotransins will inform the rational design of small molecules with improved or altered selectivity toward distinct signal peptides.

## Methods

### Chemicals and reagents

Lauryl maltose neopentyl glycol detergent was obtained from Anatrace. Superose-12 gel filtration media was obtained from GE Healthcare. Anti-Sec61α and anti-RPL16 antibodies were obtained from Abcam. Cryo-EM grids were purchased from Quantifoil Micro Tools GmbH.

### Mice

BALB/c mice (H-2d) were purchased from Taconic Biosciences. All animal studies were conducted in compliance with the NIH Guide for the Care and Use of Laboratory Animals and approved by the Kezar Life Sciences Institutional Animal Care and Use Committee.

### Arthritis model

Anticollagen antibody-induced arthritis was induced in 7- to 8-week-old female BALB/c mice (kept on breeder chow) by intravenous (IV) administration of 1.75 mg of a cocktail of five antibodies against type II collagen (Chondrex) followed by intraperitoneal challenge with 25-µg lipopolysaccharide (LPS) on day 3. Treatment was initiated after clinical signs of arthritis were observed (day 4). Paws were scored for disease severity on a 0 (no disease)—4 (maximal swelling) scoring system and summed for individual animal scores. Statistical analyses (two-way ANOVA followed by Bonferroni post hoc analysis) were performed using GraphPad Prism Software (version 7.01). Statistical significance was achieved when *P* value was less than 0.05. For efficacy studies, KZR-8445 was formulated in an aqueous solution of 10% ethanol/10% (wt/vol) Kolliphor EL and was administered three times a week (QODx3) every other day as an IV bolus. Dexamethasone was purchased from Sigma-Aldrich and administered QODx3 every other day intraperitoneally.

### Biochemical characterization of the ribosome–Sec61 complex

Sheep pancreatic ER microsomes (SRM) were isolated according to the method described earlier^48,49^. Microsomes were then resuspended in buffer containing 50 mM N-2-hydroxyethylpiperazine-N'-2-ethanesulfonic acid (HEPES) (pH 7.4), 200 mM potassium acetate (KoAc), 10 mM magnesium acetate (MgOAc) and 1 mM dithiothreitol (DTT) and treated with micrococcal nuclease in the presence of 1 mM CaCl_2_ to convert the polysomes into monosomes. The reaction was stopped by chelating the Ca^2+^ with 2 mM ethylene glycol-bis(β-aminoethyl ether)-N,N,N′,N′-tetraacetic acid (EGTA). Microsomes were aliquoted and stored at −70 °C until further use. To identify optimal conditions where inhibitor-bound Sec61 can be purified, photo-affinity labeling and click chemistry were used as previously described^15,16^. Detergent-solubilized ER microsomes containing 100-nM Sec61 were first incubated for 30 min with KZR-8445 or DMSO, then with 1-µM photo-cotransin CT7 for 10 min and crosslinking was performed by ultraviolet irradiation for 10 min. After denaturation with 1% SDS, copper-catalyzed click chemistry was used to label the crosslinked adducts with the tetramethylrhodamine (TAMRA) fluorophore. The labeled proteins were analyzed by SDS–PAGE and in-gel fluorescence followed by Western blotting using anti-Sec61α and anti-RPL18 antibodies. Using this method, several detergents and solubilization conditions were screened to find the optimal condition where the inhibitor stably remains bound.

### Ribosome–Sec61 complex purification

For the purification of the inhibitor-bound ribosome–Sec61 complexes, 50 µl of SRM were thawed and KZR-8445 was added to a final inhibitor concentration of 10 µM and incubated on ice for 30 min. LMNG was then added at a final concentration of 1% to solubilize the microsome for 60 min on ice with occasional mixing. Solubilized material was centrifuged at 21,000*g* and further purified using 1 ml Superose-12 gel filtration resin in 50 mM HEPES (pH 7.4), 200 mM KoAc, 10 mM MgoAc, 1 mM DTT, 0.003% LMNG and 1 µM KZR-8445. Ten fractions each containing approximately 100 µl sample were collected, and A_260_ absorbance was measured using a nanodrop spectrophotometer. The final concentration of the sample was estimated using the molar extension coefficient of eukaryotic ribosomes^50^. The peak fraction was supplemented to 10 µM KZR-8445 and incubated for 30 min on ice. The sample was centrifuged at 22,000 r.p.m. for 10 min to exclude any aggregates before freezing grids.

### Grid preparation and data acquisition

Holey-carbon grids (Quantifoil, R1.2/1.3 with 2 nm C) were coated with pentylamine (Sigma-Aldrich, 171409) using a method described earlier^9^ and glow-discharged with a plasma cleaner. In total, 3 µl of the freshly prepared sample at a final concentration of 300–500 nM was applied to the grid and blotted for 1.5 s before vitrification in liquid ethane precooled by liquid nitrogen. Cryo-grid preparation was assisted by an automated plunge freezer (Leica Microsystems) with the inner chamber set at 15 °C and 90% humidity. The cryo-grids were prescreened with a 200 kV FEI Talos Arctica microscope (FEI Falcon II camera). Final high-resolution datasets were collected on a 300 kV FEI Titan Krios TEM (Gatan K3 summit camera) with GIF Quantum energy filter (Gatan). The images were collected at a dose rate of 0.97e^−^ s^−1^ Å^−2^ and with an exposure time of 3 s. Movie stacks (50 frames each) were recorded under super-resolution conditions. The magnification was set at ×105,000, and the defocus ranged from −0.7 μm to −2.2 μm. Statistics for data collection are summarized in Extended Data Table 1.

### Image processing

All cryo-EM data processing was performed with Relion 3.0 and 3.1 (ref. ^51^) maintained within the Scipion 3.0.7 software package. Frames from 30,261 micrographs were aligned and motion corrected with MotionCor2 using 15 (5 × 5) patches, the default B-factor of 150 and 2x binning. Defocus values were calculated from the nondose–weighted micrographs with Gctf^11^ using information up to a resolution limit of 10 Å. A total of 31 micrographs with Gctf estimated maximum resolution worse than 20 Å were discarded. A total of 1,089,031 particles were picked from the resulting 30,230 micrographs with SPHIRE-crYOLO^12^ using a confidence threshold of 0.05 and a box size of 800 pixels. Selected particles were extracted with a binned pixel size of 3.32 Å and 2D classified to remove aberrant particles. Three rounds of 2D classification were carried out, and 30 good classes containing 729,800 particles were selected after the final round. These were then refined to generate an initial 3D reconstruction with a resolution of 6.7 Å. Alignment information from well-resolved 3D classes containing 266,968 particles was used to re-extract the particles with an unbinned super-resolution pixel size of 0.83 Å. These particles were then subjected to iterative rounds of 3D refinement and contrast transfer function (CTF) refinement until the Fourier shell correlation converged at 3.6 Å. The output particles from refinement were then 3D classified without alignment that generated ten classes. Nontranslating ribosomes were distinguished from translating ribosomes by A/P/E-site occupancy of the classes. The final map from nontranslating ribosome–Sec61 complexes (containing 136,742 particles) was then postprocessed to 3.2 Å.

### Model building and refinement

An initial model for Sec61 was built using the structure of signal peptide-engaged Sec61 (Protein Data Bank (PDB): 3JC2). The model was used to cut out the ribosome density from a map of nontranslating ribosome–Sec61-KZR-8445 particles. The ribosome density was initially removed from the map by using the Map Eraser function in UCSF ChimeraX^52^ followed by map truncation outside the Sec61 region using phenix.map_box^53^. The resulting truncated map was then used in automatic MD flexible fitting with the program Namdinator^54^. The final model was built using Coot and Phenix real-space refinement^55^. The most likely low-energy states of KZR-8445 were calculated as described^32,33^. All figures were generated using UCSF ChimeraX^56^. The most likely low-energy states of KZR-8445 were calculated using the LowModeMD Search method^57^ implemented within MOE^58^ and as previously described^32,33^.

### Luciferase reporter assay

HEK293T cells (0.3 × 10^6^) were seeded per well of a six-well plate and incubated at 37 °C in 5% CO_2_ for 24 h to adhere. Each of the two wells was transfected with 1 µg of plasmid encoding a luciferase with the signal peptide of either VCAM1 or pPL using PEI in a 5:1 ratio, after which cells were returned to the incubator for a further 24 h. Transfected cells were then resuspended following trypsinization, cell count estimated using a TC20 Automated Cell Counter (BioRad) and diluted to seed a 96-well flat-bottom plate with 1.6 × 10^4^ cells per well. Cells were returned to the incubator for 6 h to adhere, after which media was removed, cells were washed once with PBS and fresh media containing dilutions of either KZR-8445 or mycolactone A/B were added (*n* = 4). Cells were then incubated for a further 24 h, after which the luciferase-containing media were transferred to a clean 96-well plate and stored at −20 °C. Luciferase activity of the media was estimated using a Gaussia-GLOW Juice Luciferase Assay kit from PJK Biotech GmbH as per the manufacturer’s instructions. Luminescence was measured using an EnSpire Multimode plate reader (PerkinElmer). Background luminescence from media placed in a well with neither cells nor drug was subtracted from all values, and measurements were calculated as a percentage of luminescence intensity of a well expressing the appropriate luciferase construct in the absence of any drug. Curves were fitted, and IC_50_ values were calculated using GraphPad Prism 8.

### SARS-CoV-2 infections

Vero E6 cells (ATCC CRL-1586) were cultured in minimum essential media supplemented with 10% FBS (standard FBS hereafter; Gibco), 2-mM l-glutamine, 100 IU ml^−1^ of penicillin and 100 µg ml^−1^ of streptomycin, and plated on either 96-well plate (PerkinElmer; 30,000 cells per well) or on a six-well plate (200,000 cells per well) for experiments. For viability assay, cells were treated with seven concentrations of KZR-8445 in triplicates for 2 h at 37 °C and 5% CO_2_ before infection, after which the cells were infected with SARS-CoV-2 (patient isolate described in ref. ^59^) or mock infected with a multiplicity of infection of 0.03 for 48 h. Supernatant samples were collected, and a cell viability assay was performed in a 96-well plate using CellTiter-Glo 2.0 cell viability assay (Promega) and Hidex Sense reader (Hidex) in a BSL-3 facility. Infectious virus amount was determined by end-point titration assay in quadruplets of each replicate and presented as TCID50 per milliliter^60^. Shortly, 10-fold dilutions of the samples were inoculated to Vero E6 cells, incubated for 5 d, fixed with 10% formaldehyde for 30 min at room temperature and stained with crystal violet. For reverse transcription (RT)–PCR, RNA was extracted from cell culture supernatant using QIAamp Viral RNA Mini Kit (Qiagen), and quantitative RT–PCR was performed using primers and probe specific to SARS-CoV-2 RdRP, as described in ref. ^61^. Emetine was used as a control as it has been described to inhibit SARS-CoV-2 transcription^62^. For protein analysis, cells were treated with five concentrations of KZR-8445 2 h before infection. At 48 h postinfection, cell samples were collected in Laemmli sample buffer (LSB; Sigma-Aldrich), and supernatants were ultracentrifuged through a 30% sucrose cushion at 141,000*g* for 90 min and resuspended in LSB. Both sample types were subjected to SDS–PAGE in 4–15% Mini-PROTEAN TGX gels (BioRad) and transferred onto nitrocellulose membranes. SARS-CoV-2 spike and nucleocapsid proteins were visualized by rabbit anti-receptor binding domain (RBD) (1 µg ml^−1^) and anti-N (200 ng ml^−1^) primary^63^, as well as IRDye 800CW Goat anti-Rabbit IgG and IRDye 680CW Goat anti-Rabbit IgG secondary antibodies (Li-Cor).

### Generation of stable Sec61 mutant cell lines

WT canine Sec61 was cloned into pcDNA5/FRT/TO (Thermo Fisher Scientific), and point mutations were generated using PCR. All mutants were sequence verified. HEK293 Flp-In T-REx cells (Thermo Fisher Scientific, R78007) were cultured in Dulbecco’s modified Eagle’s medium (Gibco) supplemented with 10% FBS (Gibco) at 37 °C in a humidified 5% CO_2_ atmosphere. Approximately 0.3 µg of pOG44 and 1 µg of targeting construct plasmids were mixed in 250 µl of OptiMEM (Thermo Fisher Scientific, 51985-026). A total of 3 µl (3 µg) Lipofectamine3000 transfection reagent was then added to the DNA mix and incubated at room temperature for 20 min, and the whole mixture was added to the cells. Cells were exposed to 100 µg ml^−^^1^ hygromycin B (Invitrogen) and 10 µg ml^−^^1^ blasticidin for 3–4 weeks until the appearance of resistant colonies. Colonies were expanded into T75 flasks and induced with doxycycline (Sigma-Aldrich, D9891) at a concentration of 1–5 µg ml^−^^1^ for 48 h. Cells were collected, and total RNA was extracted followed by cDNA amplification and sequencing of Sec61 with gene-specific PCR primers to confirm. Cells were then used for the luciferase reporter assays as above.

### CellTiter-Glo (CTG) cell viability assays

PBMCs were isolated from fresh whole blood (AllCells) via Leucosep tube (Greiner Bio-One), including red blood cell lysis using Pharm Lyse solution (BD Biosciences). For viability experiments, unstimulated PBMCs were plated at 200,000 cells per well in 100 µl growth media (RPMI 1640 supplemented with 5% FBS, 2 mM l-glutamine, 10 mM HEPES and 100 IU penicillin/100 µg ml^−^^1^ streptomycin) in 96-well clear polystyrene round-bottom tissue culture-treated plates. A total of 50 µl of growth media and 50 µl of 4× compound stock solution (7-point log dilutions, final concentration range of 0.025–25,000 nM, 0.25% DMSO) were immediately added, and the cells were cultured at 37 °C with 5% CO_2_ for 24 h. Subsequently, plates were centrifuged at ~500*g* for 5 min at room temperature, and 100 µl of supernatant was removed for subsequent cytokine analysis. For viability determination, 100 µl of CTG (Promega) was added to the remaining 100 µl cells or growth media. Plates were shaken for 2 min, and the reaction was transferred to a black-wall, clear flat-bottom 96-well polystyrene plate for assay readout. The assay plate was allowed to sit statically for 10 min at room temperature before measuring luminescence using an M1000 Pro plate reader (Tecan).

### Cytokine secretion assays

For cytokine experiments, human PBMCs and mouse splenocytes were either left unstimulated or stimulated with lipopolysaccharide or antibodies against CD3 and CD28. PBMCs were isolated as above; splenocytes were obtained from 8- to 12-week-old female BALB/c mice. Spleens were collected into cold PBS supplemented with 100 IU penicillin and 100 µg ml^−^^1^ streptomycin and then kept on ice until tissue disruption/cell isolation was carried out using a 100 µm nylon cell strainer (BD Biosciences). Strained tissue was centrifuged at ~300*g* for 5 min at 4 °C, and then red blood cells were lysed in Pharm Lyse solution (BD Bioscience), before being rinsed in PBS and resuspended in growth media (RPMI 1640 supplemented with 10% FBS, 1 mM sodium pyruvate, 100 IU penicillin, 100 µg ml^−^^1^ streptomycin and 0.05 mM β-mercaptoethanol).

For anti-CD3/CD28-stimulation, 96-well clear polystyrene round-bottom tissue culture-treated plates were coated overnight at 4 °C with 100 µl per well of 2 µg ml^−^^1^ mouse antihuman CD3e (Thermo Scientific Fisher, MA1-10176; clone OKT3 for PBMCs) or 5 µg ml^−^^1^ hamster antimouse CD3e (BD Biosciences, 553057; clone 145-2C11 for splenocytes). Immediately before cell plating, anti-CD3-coated wells were emptied and washed twice with 200 µl PBS. PBMCs and splenocytes were plated at a density of 200,000 cells per well in 100 µl of their respective growth media. A total of 50 µl of growth media (for unstimulated cells), 50 µl of 4x LPS (for LPS-stimulated cells; Sigma-Aldrich, L431; final concentration 1 µg ml^−^^1^ for PBMCs, 5 µg ml^−^^1^ for splenocytes), or 50 µl of 4x anti-CD28 (for anti-CD3/CD28-stimulated cells; final concentration 2 µg ml^−^^1^ for PBMCs, 5 µg ml^−^^1^ for splenocytes) were immediately added to cells, along with 50 µl of 4x compound stock (7-point log dilutions, final concentration range 0.025–25,000 nM, 0.25% DMSO). Mouse antihuman CD28 (for PBMCs) was from BD Biosciences (555725, clone CD28.2) and hamster antimouse CD28 (for splenocytes) was from BD Biosciences (553294, clone 37.51). Cells were cultured as above for 24 h, and the supernatant was collected for cytokine analysis.

Cytokine concentrations in 100 µl media supernatant were quantified using an MSD U-PLEX electrochemiluminescent immunoassay (Meso Scale Diagnostics, K15067L for human and K15069L for mouse). Biomarker assays were custom 96-well 7-plex plates for simultaneous analysis of species-specific GM-CSF, IFNg, IL-1β, IL-2, IL-6, IL-23 and TNFα. PBMC supernatant was diluted 1:10 in assay diluent before analysis; splenocyte supernatant was assayed neat or diluted 1:4 or 1:10. Assays (including calibrator standard curve generation) were performed according to the manufacturer’s instructions and were read on a MESO QuickPlex SQ 120 imager. Compound IC_50_s were calculated using four-parameter logistic regression of DMSO-normalized dose–response curves; LPS-stimulation conditions were used for the calculation of IL-1β, IL-6 and IL-23 IC_50_s; anti-CD3/CD28-stimulation for GM-CSF, IFNg, IL-2 and TNFα IC50 values.

### MD simulations

Sec61 with the modeled KZR-8445 inhibitor was embedded in a multicomponent lipid membrane consisting of 54% 1-palmitoyl-2-oleoyl-*sn*-glycero-3-phosphocholine (POPC), 21% 1-palmitoyl-2-oleoyl-*sn*-glycero-3-phosphoethanolamine (POPE), 10% 1-palmitoyl-2-oleoyl-*sn*-glycero-3-phosphoinositol (POPI), 4% 1-palmitoyl-2-oleoyl-*sn*-glycero-3-phospho-L-serine (POPS), 4% N-palmitoylsphingomyelin (PSM) and 7% cholesterol, mimicking the known ER membrane lipid composition^64–67^. The unresolved loops of Sec61α (residues 46–61, 98–104, 135–146, 221–228 and 312–336) were truncated to maintain a continuous chain (residues 11–466). The Sec61β that makes no contact with KZR-8445 was modeled as all-Ala due to the uncertainty in the residue assignment. The membrane, consisting of 500 lipids, was solvated with 40,000 water molecules, 135 mM KCl and neutralizing counter ions. Five independent starting configurations were generated using CHARMM-GUI^68,69^ with protein positioned using PPM 2.0 (ref. ^70^). Three systems without KZR-8445 and one with an N300A mutant were set up similarly. KZR-8445 was parameterized within CHARMM-GUI^71^. The mutually compatible force fields, CHARMM36m (protein)^72,73^, CHARMM36 (lipids)^74^, CGenFF with a positive dummy particle for bromobenzyl sigma hole (inhibitor)^75,76^ and TIP(S)3P (water) were used^77,78^.

All systems were first subjected to minimization and equilibration protocols^79^, after which each was simulated for 1 µs. GROMACS 2021 was used to perform all simulations^80^ with recommended parameters^79^. The leap-frog integrator was used with a time step of 2 fs. Buffered Verlet lists were used^81^. The Lennard-Jones forces were switched to zero between 1.0 and a cutoff distance of 1.2 nm. Long-range electrostatic interactions were included by the smooth particle mesh Ewald algorithm^82,83^. Temperatures of the protein (including the inhibitor), the lipids and the solvent (water and ions) were separately coupled to a Nosé–Hoover thermostat^84,85^, with a target temperature of 310 K and a relaxation time of 1 ps. The pressure was maintained at 1 bar with a semi-isotropic Parrinello–Rahman barostat^86^. The target pressure was set to 1 bar, the compressibility to 4.5 × 10^–5^ bar^−1^ and the relaxation time constant to 5 ps. Bonds involving hydrogens were constrained with p-LINCS^87,88^.

Conformational stabilities of Sec61 and KZR-8445 were evaluated with root mean squared deviations from the Sec61 backbone and KZR-8445 as a function of simulation time for each replica using *gmx rms*. For equilibrium properties, we accumulated the trajectories after discarding the first 200 ns of each replica (5 × 800 ns = 4 µs and 3 × 800 ns = 2.4 µs for systems with and without KZR-8445) and extracted root mean square fluctuations (RMSF) of Sec61 backbone and KZR-8445 with *gmx rmsf*, the distributions of the distance between lateral gate helices with *gmx distance*, hydrogen bond occupancies with the *Hbonds* plugin in the program VMD^89^ (donor–acceptor cutoff of 3.5 Å and hydrogen–donor–acceptor angle of 30°) and the interaction energies of various Sec61α residues with KZR-8445 with energy groups and the *rerun* functionality of *gmx mdrun*. The snapshot of the simulation system was rendered using the Tachyon renderer in VMD^89^. The stability of the inhibitor at the binding site (WT versus N300A mutant) was evaluated with the time evolution of the distance between the key hydrogen bonding partners, N300 (or A300 in the mutated Sec61) and the peptide bond hydrogen between side chains 3 and 4 in KZR-8445. The minimum distance between these binding partners was calculated using the *gmx mindist* tool for the N300A mutant as well as the five WT replica simulations.

### Reporting summary

Further information on research design is available in the Nature Portfolio Reporting Summary linked to this article.

## Online content

Any methods, additional references, Nature Portfolio reporting summaries, source data, extended data, supplementary information, acknowledgements, peer review information; details of author contributions and competing interests and statements of data and code availability are available at 10.1038/s41589-023-01326-1.

### Supplementary information

Supplementary Information Supplementary Note.

Reporting Summary

Supplementary Video 1 Cryo-EM density assignment.

Supplementary Video 2 Ligand interaction site.

Supplementary Video 3 Comparing KZR-8445-engaged and idle-Sec61α.

Supplementary Video 4 Comparing KZR-8445- and a-factor SP-engaged Sec61α.

### Source data

Source Data Fig. 1 Statistical source data.

Source Data Fig. 3 Statistical source data.

Source Data Fig. 4 Statistical source data.

Source Data Extended Data Fig. 1 and Table 1 Statistical source data.

Source Data Extended Data Fig. 1 Unprocessed western blots and gels.

Source Data Extended Data Fig. 2 and Table 2 Statistical source data.

Source Data Extended Data Fig. 2 Unprocessed western blots and gels.

Source Data Extended Data Fig. 4 and Table 4 Statistical source data.

Source Data Extended Data Fig. 6 and Table 6 Statistical source data.

Source Data Extended Data Fig. 7 and Table 7 Statistical source data.

## Figures and Tables

**Extended Data Fig. 8:**
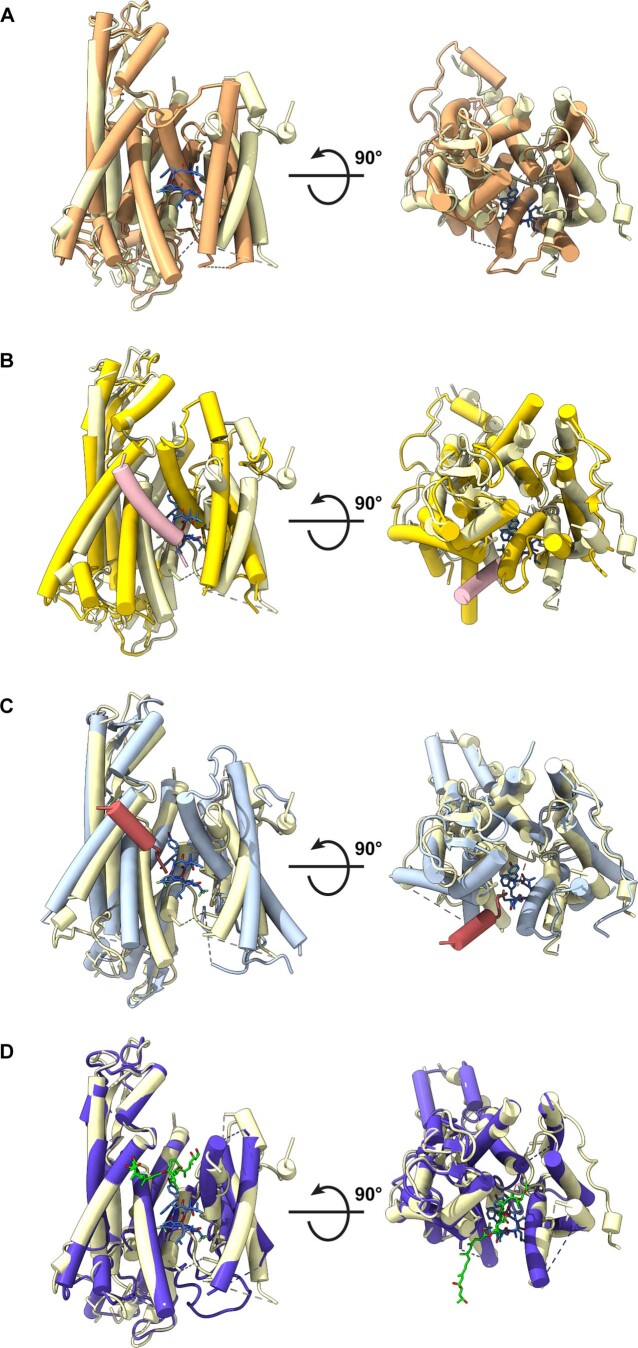
Comparison of different structural states of mammalian Sec61 translocon. **(a–d)** Comparisons of Sec61α (wheat) with bound KZR-8445 (royal blue) and **(a)** ‘idle’ mammalian Sec61α (PDB:3J7Q, orange), **(b)** mammalian Sec61 (PDB:3JC2, yellow) engaged with the preprolactin signal peptide (pink), **(c)** yeast Sec61 (PDB:7AFT, pale blue) engaged with the prepro-α factor signal peptide (red), or **(d)** mammalian Sec61α (PDB:6Z3T, green) with bound mycolactone (purple).

**Extended Data Table 1:**
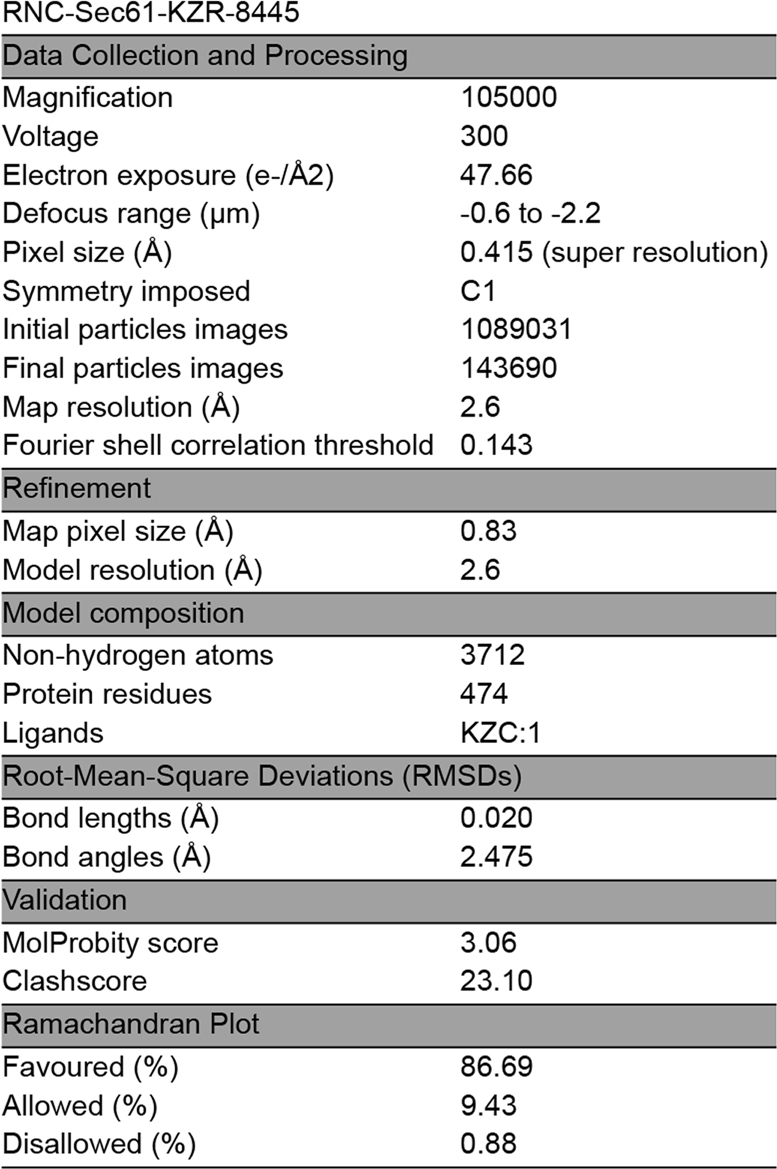
Refinement and model statistics

Cryo-EM refinement and model statistics.

## Data Availability

Coordinates of the KZR-8445-bound structure of the mammalian translocon and the corresponding cryo-EM density map have been deposited to Protein Data Bank accession code PDB-7ZL3 and Electron Microscopy Data Bank accession code EMD-14776, respectively. The cryo-EM micrograph data have been deposited to the EMPIAR Data Bank under the code EMPIAR-11405. Simulation data are available in the Zenodo repository at DOI: 10.5281/zenodo.7303653. The datasets generated and/or analyzed during the current study are attached.
