# Supplementary material for: Signal peptide mimicry primes Sec61 for client-selective inhibition
Source: Nat Chem Biol. 2023 May 11;19(9):1054–62. doi: 10.1038/s41589-023-01326-1 (PMC10449633; doi:10.1038/s41589-023-01326-1)
Supplement: Supplementary file 1 — Supplementary Note. [file 41589_2023_1326_MOESM1_ESM.pdf]

nature chemical biology

Article

https://doi.org/10.1038/s41589-023-01326-1

Signal peptide mimicry primes Sec61 for client-selective inhibition

In the format provided by the authors and unedited

SUPPLEMENTARY NOTE

Experimental Procedures and Characterization data for KZR-8445 and KZR-9508 1
Nα-(tert-Butoxycarbonyl)-Nα-methyl-L-tryptophan 1
1-(4-Bromobenzyl)-Nα-(tert-butoxycarbonyl)-Nα-methyl-L-tryptophan 2
Nα-(((9H-Fluoren-9-yl)methoxy)carbonyl)-1-(4-bromobenzyl)-Nα-methyl-L-tryptophan 2
Allyl L-leucinate 3
Allyl Nα-(((9H-fluoren-9-yl)methoxy)carbonyl)-1-(4-bromobenzyl)-Nα-methyl-L-tryptophyl-L-leucinate 3
Deprotection Allyl esters 3
General procedures for peptide elongation on resin 4
Resin loading: 4
Resin washing method A: 5
Resin washing method B: 5
Resin washing method C: 5
Fmoc removal: 5
Resin coupling method A: 6
Resin coupling method B: 6
Resin coupling method C: 6
Synthesis of the linear peptide 7
Cleavage from resin and isolation of heptadepsipeptide hydrochloride: 7
Macrocyclization method A 8

Supplementary Note: Procedures and Characterization data for KZR-8445 and KZR-9508

NMR spectra were recorded on a Bruker spectrometer at 400 MHz. Chemical shifts were reported as parts per million (ppm) from an internal tetramethylsilane standard or solvent references. LCMS was performed on a Waters Alliance HT LC/MS (0.2 mL/min) S8 using an Xterra MS C18 column (Waters) and a water/acetonitrile gradient (0.1% formic acid). Preparative HPLC was performed on a Waters 2545 binary gradient module with a Waters 2998 photodiode array module and using an Atlantis T3 Prep ODB 5 μM 19 × 250 mm C18 column. Analytical thin-layer chromatography was performed with silica gel 60 F254 glass plates (EM Science). Silica gel chromatography was performed with 230-400 mesh silica gel. Flash chromatography was performed using a Teledyne ISCO combiFlash Rf system. All solvents were of ACS grade (Fisher Scientific) and used without further purification. Commercially available reagents were used without further purification.

N$^{\alpha}$-(*tert*-Butoxycarbonyl)-N$^{\alpha}$-methyl-L-tryptophan

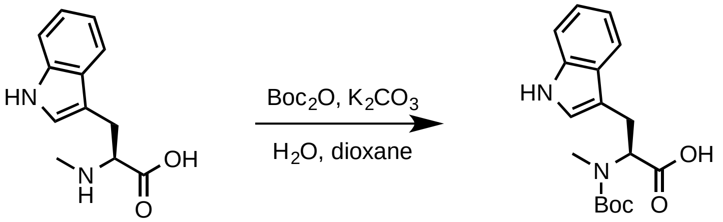

To a suspension of abrine (2.00 g, 9.16 mmol) in water (17 mL) and dioxane (17 mL) was added K_2CO_3 (3.60 g, 26.12 mmol) and then, dropwise, a solution of Boc_2O (2.40 g, 11.00 mmol) in dioxane (10 mL). The mixture was stirred at room temperature for 12 h then concentrated to remove dioxane. The reaction mixture was washed with hexane (2 × 20 mL) then acidified to pH ~ 3 with citric acid (10% aqueous solution) and extracted with EtOAc (2 × 20 mL). The combined organic phase was washed with water (10 mL), brine (10 mL), then dried with (Na_2SO_4), filtered and concentrated under reduced pressure to afford N$^{\alpha}$-(*tert*-butoxycarbonyl)-N$^{\alpha}$-methyl-L-tryptophan (2.76 g, 95%) as a colorless solid. **^{1}H-NMR** (400 MHz; $CDCl_3$, 2 rotamers): δ 8.14 (broad m, 1H), 7.64 (d, *J* = 7.8 Hz, 1H), 7.39 (d, *J* = 8.0 Hz, 1H), 7.23 (dd, *J* = 7.9, 7.2 Hz, 1H), 7.16 (t, *J* = 7.4 Hz, 1H), 7.09 (s, 0.5), 7.04 (s, 0.5H), 4.91-4.86 (m, 1H), 3.53-3.41 (m, 1H), 3.41-3.36 (m, 0.5H), 3.24-3.17 (m, 0.5H), 2.83 (s, 1.6 H), 2.74 (s, 1.4 H), 1.47 (s, 4.5H), 1.24 (s, 4.5H). **HRMS** (ESI): Calculated for $C_{17}H_{21}N_2O_4$ $[M-H]^-$, 317.1507; Found, 317.1529.

1-(4-Bromobenzyl)-N$^{\alpha}$-(*tert*-butoxycarbonyl)-N$^{\alpha}$-methyl-L-tryptophan

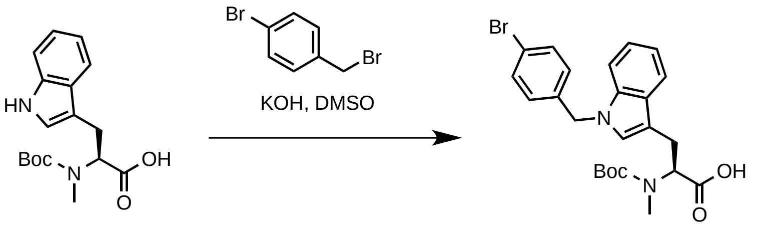

To a stirred solution of freshly powdered potassium hydroxide (4.32 g, 75.38 mmol) in dimethyl sulfoxide (anhydrous, 37 mL) at room temperature under argon was added N$^{\alpha}$-(*tert*-butoxycarbonyl)-N$^{\alpha}$-methyl-L-tryptophan (6.00 g, 18.85 mmol) and the mixture was stirred for 1 h. 4-Bromobenzyl bromide (5.18 g, 20.73 mmol) was then added and the mixture was stirred under argon for 16 h. The solution was diluted with water (10 mL), washed with diethyl ether (2 × 5 mL), and then acidified with citric acid (10% aqueous solution) until pH ~ 3. The mixture was extracted with EtOAc (3 × 20 mL) and the combined fractions were washed with water (10 mL) then brine (10 mL) then dried (Na_2SO_4) and concentrated under reduced pressure to afford 1-(4-bromobenzyl)-N$^{\alpha}$-(*tert*-butoxycarbonyl)-N$^{\alpha}$-methyl-L-tryptophan (7.61 g, 83%) as a pale yellow solid. **^{1}H-NMR** (400 MHz; $CDCl_3$): δ 7.65 (d, *J* = 7.6 Hz, 1H), 7.51 (d, *J* = 8.4 Hz, 1H), 7.44-7.41 (m, 2H), 7.21 (dd, *J* = 6.1, 1.2 Hz, 2H), 6.95 (d, *J* = 8.4 Hz, 2H), 5.24 (s, 2H), 4.89-4.82 (m, 1H), 3.49-3.40 (m, 2H), 3.24-3.20 (m, 1H), 2.82 (s, 1.5H), 2.70 (s, 1.5H), 1.43 (s, 4.5H), 1.23 (s, 4.5H). **LCMS** (ESI): $[M-H]^-$, 486.1.

N$^{\alpha}$-(((9H-Fluoren-9-yl)methoxy)carbonyl)-1-(4-bromobenzyl)-N$^{\alpha}$-methyl-L-tryptophan

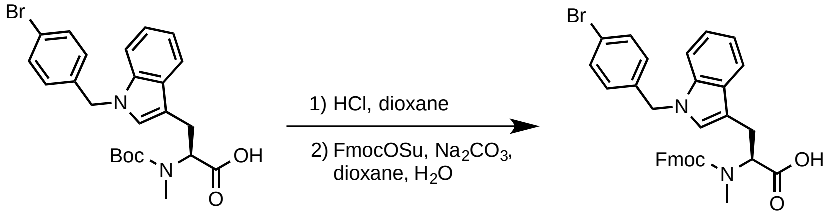

To a solution of 1-(4-bromobenzyl)-N$^{\alpha}$-(*tert*-butoxycarbonyl)-N$^{\alpha}$-methyl-L-tryptophan (2.50 g, 5.13 mmol) in dioxane (1 mL) was added HCl (5 mL of a 4 M solution in dioxane). The mixture was stirred for 2 h at room temperature then concentrated under reduced pressure, co-evaporated with dioxane several times then redissolved in a mixture of dioxane (12 mL) and water (12 mL). To this mixture was added Na_2CO_3 (1.25 g, 11.80 mmol) followed by FmocOSu (2.25 g, 6.67 mmol) and the mixture was stirred vigorously overnight. The mixture was concentrated to remove dioxane, acidified with citric acid (10 mL of a 10% aqueous solution) and extracted with EtOAc (3 × 10 mL) the combined organic fractions were washed with water, then brine, dried ($MgSO_4$), filtered, and concentrated under reduced pressure. The residue was subjected to flash chromatography (silica, gradient elution, 0-5% MeOH/DCM) to afford N$^{\alpha}$-(((9H-fluoren-9-yl)methoxy)carbonyl)-1-(4-bromobenzyl)-N$^{\alpha}$-methyl-L-tryptophan (2.83 g, 91%) as a

pale yellow solid. **^{1}H-NMR** (400 MHz; $CDCl_3$, rotamers): δ 7.80-7.73 (m, 3H), 7.67-7.60 (m, 1H), 7.54 (t, *J* = 6.8 Hz, 1H), 7.49-7.39 (m, 5H), 7.36-7.31 (m, 2H), 7.22-7.12 (m, 4H), 6.97 (s, 1H), 6.91-6.86 (m, 2H), 5.18-5.15 (m, 2H), 5.05-5.01 (m, 0.5H), 4.87-4.82 (m, 0.5), 4.51-4.35 (m, 2H), 4.26-4.17 (m, 1.5H), 4.05-4.01 (m, 0.5), 3.56-3.50 (m, 1H), 3.41-3.34 (m, 1H), 2.86 (s, 3H). **LCMS** (ESI): $[2M-H]^-$, 1218.2.

Allyl L-leucinate

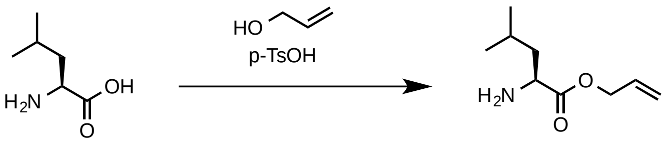

To a solution of L-leucine (2.50 g, 19.06 mmol) in allyl alcohol (25 mL) was added p-TsOH•H_2O and the mixture was stirred at 90 °C (oil bath) for 16 h. The next day the solution was concentrated under reduced pressure to a solid residue, which was dissolved in DCM (25 mL) and washed with $NaHCO_3$ (10 mL of a saturated aqueous solution). The aqueous fraction was extracted with DCM (2 × 10 mL) and the combined organics were dried (Na_2SO_4), and filtered directly into HCl (4 equiv, ~ 19 mL of a 4 M solution in dioxane). The solution was concentrated under reduced pressure then precipitated with Et_2O. The mixture was briefly sonicated, left to settle for a few minutes, and the solution was decanted from the solid using a pipette. The product was dried under vacuum overnight, affording the title compound (3.07 g, 77%) as a pale yellow solid. **^{1}H-NMR** (400 MHz; DMSO-d_6): δ 8.61 (t, *J* = 0.4 Hz, 3H), 5.94 (ddt, *J* = 17.2, 10.6, 5.4 Hz, 1H), 5.39 (dq, *J* = 17.3, 1.6 Hz, 1H), 5.29 (dq, *J* = 10.5, 1.3 Hz, 1H), 4.70 (d, *J* = 5.4 Hz, 2H), 4.01-3.98 (m, 1H), 1.81-1.73 (m, 1H), 1.67 (t, *J* = 7.3 Hz, 2H), 0.91 (dd, *J* = 6.5, 1.2 Hz, 6H).

Allyl N$^\alpha$-(((9H-fluoren-9-yl)methoxy)carbonyl)-1-(4-bromobenzyl)-N$^\alpha$-methyl-L-tryptophyl-L-leucinate

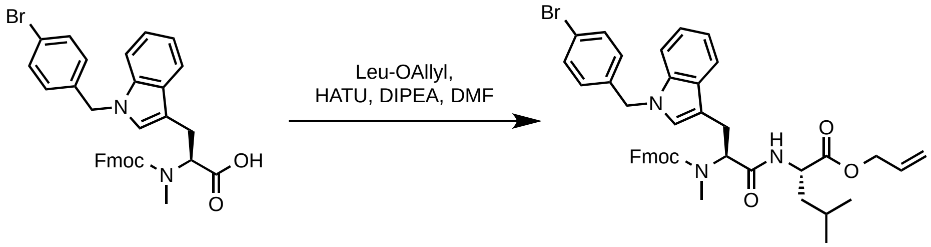

A 100 mL round bottom flask was charged with N$^\alpha$-(((9H-fluoren-9-yl)methoxy)carbonyl)-1-(4-bromobenzyl)-N$^\alpha$-methyl-L-tryptophan (6.50 g, 10.66 mmol), Leu-OAllyl (2.54 g, 12.26 mmol) and HATU (4.87 g, 12.87 mmol). The mixture was dissolved in DMF (50 mL) under argon and cooled to 0 °C, then DIPEA (5.57 mL, 32.00 mmol) was added and the mixture was stirred for 2 h, diluted with EtOAc (50 mL) and washed with water (4 × 20 mL). The combined aqueous phases were extracted

again with EtOAc (10 mL) and the combined organics were washed with brine (10 mL) and dried (Na_2SO_4), filtered and concentrated to afford Allyl N^{α}-(((9H-fluoren-9-yl)methoxy)carbonyl)-1-(4-bromobenzyl)-N^{α}-methyl-L-tryptophyl-L-leucinate (7.92 g, 97%) a yellow solid. **^{1}H-NMR** (400 MHz; $CDCl_3$, rotamers): δ 7.80-7.79 (m, 2H), 7.73-7.63 (m, 2H), 7.55-7.34 (m, 5H), 7.18 (d, *J* = 0.1 Hz, 3H), 7.00 (s, 1H), 6.90-6.88 (m, 2H), 6.45-6.43 (m, 1H), 6.04-5.86 (m, 1H), 5.38-5.07 (m, 4H), 4.68-4.60 (m, 3H), 4.44-4.35 (m, 1H), 4.23-4.14 (m, 1H), 3.93-3.80 (m, 1H), 3.52-3.43 (m, 1H), 3.28-3.22 (m, 1H), 2.94-2.90 (m, 3H), 1.69-1.51 (m, 4H), 0.99-0.88 (m, 6H). **LCMS** (ESI): $[M+H]^+$, 764.6.

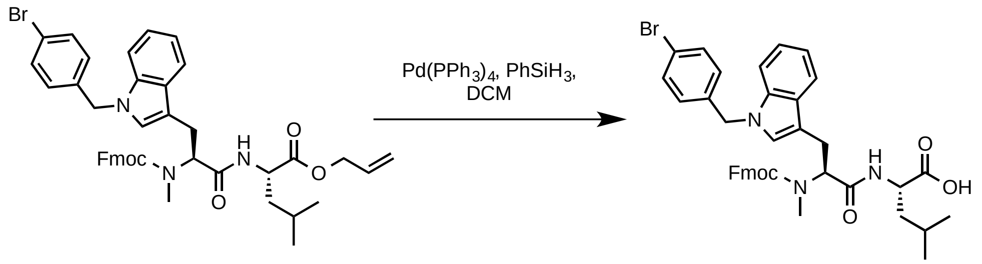

To a solution of the allyl ester (8.29 g, 10.87 mmol) in DCM (75 mL) at room temperature under argon was added $PhSiH_3$ (2.62 mL, 21.74 mmol) followed by $Pd(PPh_3)_4$ (628 mg, 0.54 mmol). The mixture was stirred for 2 h then concentrated onto silica and chromatographed (silica, 0-5%, MeOH/DCM) to afford the carboxylic acid (7.39 g, 94%) as a brown solid. **^{1}H-NMR** (400 MHz; $CDCl_3$, rotamers): δ 7.79 (dd, *J* = 7.2, 0.6 Hz, 2H), 7.66 (d, *J* = 1.4 Hz, 3H), 7.50-7.39 (m, 6H), 7.18-7.15 (m, 3H), 6.97 (d, *J* = 0.3 Hz, 1H), 6.87-6.85 (m, 2H), 5.18-5.06 (m, 3H), 4.60-4.54 (m, 1H), 4.39-4.31 (m, 1H), 4.21-4.14 (m, 1H), 3.54-3.40 (m, 2H), 3.25-3.18 (m, 1H), 2.94-2.84 (m, 3H), 1.72-1.50 (m, 3H), 0.94-0.87 (m, 6H). **LCMS** (ESI): $[M+H]^+$, 722.6.

General procedures for peptide elongation on resin

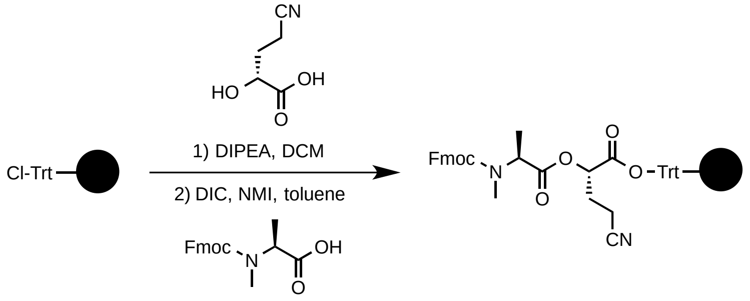

Resin loading:

A 50 mL Luer lock polypropylene syringe, fitted with a Teflon stopcock and internal polypropylene frit was charged with Cl-2-Cl-Trityl resin (2.5 g, Bachem, 1.6 meq/g, 4.07 mmol). The stopcock was closed, DCM (20 mL, anhydrous) was added and the syringe was fitted with a polypropylene plunger and

agitated, end-over-end, for 1 h to swell the resin. The resin was filtered under suction and a solution of (2*R*)-4-cyano-2-hydroxybutanoic acid (789 mg, 6.11 mmol, 1.5 equiv) and DIPEA (2.11 mL, 12.23 mmol, 3 equiv) in DCM (20 mL, anhydrous) was added and the mixture was agitated, end-over-end, for 16 h. The resin was then filtered under suction and washed with DCM (2 × 20 mL × 1 min gentle shaking) then with toluene (anhydrous, 2 × 20 mL × 1 min gentle shaking). The resin was filtered under suction and a premixed solution of Fmoc-*N*-Me-Ala-OH (3.58 g, 11.0 mmol, 2.0 equiv), *N*,*N*-diisopropyl carbodiimide (1.72 mL, 11.0 mmol, 2 equiv) and *N*-methylimidazole (0.88 mL, 11.0 mmol, 2 equiv) in toluene (20 mL, anhydrous) was added and the mixture was agitated, end-over-end, for 1 h. The resin was drained and the coupling procedure was repeated once more. The resin was then washed using resin 'washing method A' described below. The resin was then dried under high vacuum overnight affording dried resin (3.59 g, 60%). Resin loading was also assessed by measuring absorbance at 290 nm of a solution prepared by treating a 1 mg sample of resin with 3 mL of a 20% 4-methylpiperidine solution in DMF for 10 min. The measured quantity of dibenzofulvene adduct was determined using a standardized calibration plot. Using this method, the measured loading was 0.96 mmol/g (60%).

Resin washing method A:

To the resin is added DMF (10 mL/g resin) and the resin is agitated by gently shaking for about 1 min. The resin is then drained under light vacuum and the procedure is repeated with *i*-PrOH (1 × 10 mL/g resin), then DMF (1 × 10 mL/g resin), then *i*-PrOH (1 × 10 mL), then DMF (1 × 10 mL/g resin), then DCM (3 × 10 mL/g resin).

Resin washing method B:

To the resin is added DMF (10 mL/g resin) and the resin is agitated by gently shaking for about 1 min. The resin is then drained under light vacuum and the procedure is repeated with *i*-PrOH (1 × 10 mL/g resin), then DMF (1 × 10 mL/g resin), then *i*-PrOH (1 × 10 mL/g resin), then DMF (3 × 10 mL/g resin).

Resin washing method C:

To the resin is added DMF (10 mL/g resin) and the resin is agitated by gently shaking for about 1 min. The resin is then drained under light vacuum and the procedure is repeated with *i*-PrOH (1 × 10 mL/g resin), then DMF (1 × 10 mL/g resin), then *i*-PrOH (1 × 10 mL/g resin), then DMF (1 × 10 mL/g resin), then toluene (3 × 10 mL/g resin).

Fmoc removal:

A solution of 4-methylpiperidine in DMF (20%, 10 mL/g resin) is added to the syringe containing the

DMF (or DCM)-swelled resin. The syringe is capped with a polypropylene plunger and the mixture was agitated, end-over-end, for 5 min. The resin is filtered under suction and the procedure repeated twice more. The resin is then drained under light vacuum and washed using ‘resin washing method B’.

Resin coupling method A:

To a solution of Fmoc-AA-OH (2 equiv) and HATU (2 equiv) in DMF (0.1 M) is added DIPEA (4 equiv) and the solution is quickly mixed to homogeneity and added to the syringe containing DMF-swelled resin. The syringe is capped with a polypropylene plunger and the mixture is agitated, end-over-end, for 1 h at room temperature, then drained under light vacuum and washed using ‘resin washing method B’. Chloranil staining assessed complete coupling: To ~0.5 mg of wet resin is added 20% acetaldehyde in DMF (100 µL) followed by 20 mg/mL chloranil in DMF (100 µL) and the beads are incubated for 2-5 min. Colorless beads indicated quantitative coupling for each step.

Resin coupling method B:

To a solution of Fmoc-AA-OH (2 equiv.) and EEDQ (2 equiv.) in toluene (0.35 M) and the solution is quickly mixed to homogeneity and added to the syringe containing toluene-washed resin. The syringe is capped with a polypropylene plunger and the mixture is agitated, end-over-end, for 2 h at room temperature, then drained under light vacuum and washed using ‘resin washing method B’. Chloranil staining assessed complete coupling: To ~0.5 mg of wet resin is added 20% acetaldehyde in DMF (100 µL) followed by 20 mg/mL chloranil in DMF (100 µL) and the beads are incubated for 2-5 min. Colorless beads indicated quantitative coupling for each step.

Resin coupling method C:

A solution of Fmoc-AA-OH (2 equiv.) and EEDQ (2 equiv.) in toluene (0.35 M) was sonicated to a thick gel. This gel was dissolved in DMF (final concentration 0.117 M). The solution was added to the syringe containing toluene-washed resin. The syringe is capped with a polypropylene plunger and the mixture is agitated, end-over-end, for 2 h at room temperature, then drained under light vacuum and washed using ‘resin washing method B’. Chloranil staining assessed complete coupling: To ~0.5 mg of wet resin is added 20% acetaldehyde in DMF (100 µL) followed by 20 mg/mL chloranil in DMF (100 µL) and the beads are incubated for 2-5 min. Colorless beads indicated quantitative coupling for each step.

Synthesis of the linear peptide

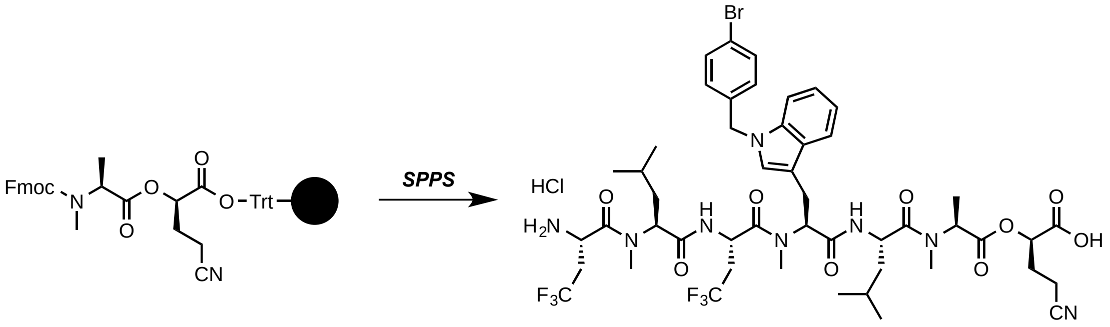

To dry Fmoc-N-Me-Ala-DGCN-loaded resin (3.00 g, 2.46 mmol) in the polypropylene syringe, fitted with a frit and stopcock, was added DCM (25 mL) and the syringe was capped with plunger and the resin was swelled by agitating, end-over-end, for 1 h. The resin was then drained and Fmoc group was removed using 'Fmoc removal' procedure. The resin was then washed using 'resin washing method C' then coupled with Fmoc-N-Me-Trp(4-BrBn)-Leu-OH using 'resin coupling method B', and washed with 'resin washing method B', then Fmoc was removed using 'Fmoc removal' procedure and resin washed with 'resin washing method B'. The peptide was elongated in a similar manner using:

1) Fmoc-Gly(CH_2CF_3)-OH using 'resin coupling method C'
2) Fmoc-N-Leu-OH using 'resin coupling method A'
3) Fmoc-Gly(CH_2CF_3)-OH using 'resin coupling method C'

After removal of the last N-terminal Fmoc group, the resin was washed using 'washing method A'.

Cleavage from resin and isolation of heptadepsipeptide hydrochloride:

To the syringe containing the washed heptadepsipeptide-loaded resin (assumed 2.46 mmol) obtained above was added HFIP (15 mL of a 20% solution in DCM) and the syringe was capped with a plunger and agitated, end-over-end, for 15 min (the resin turned red). Using a light vacuum, the solution was eluted into a 250 mL round bottom flask containing HCl (0.25 M solution in EtOAc, 40 mL ~4 equiv). The cleavage procedure was repeated once more and the solution was collected in the same flask of HCl. The remaining resin was rinsed into the HCl solution with DCM (2 × 10 mL) and the combined solution was concentrated under reduced pressure. The residue was dissolved in a minimal volume of EtOAc and Et_2O was added and was accompanied by precipitation of a white solid. The mixture was sonicated for ~10 seconds and the precipitate left to settle for a few minutes before the liquid was carefully removed using a pipette. The residual solid was triturated with Et_2O and dried under high vacuum to afford the heptadepsipeptide hydrochloride (930 mg, 33%) a white solid. The product was

used in the next step without purification. **HRMS** (ESI): Calculated for $[M-H]^-$ $C_{53}H_{87}N_8O_9$, 1101.3878; Found, 1101.3868.

Macrocyclization method A

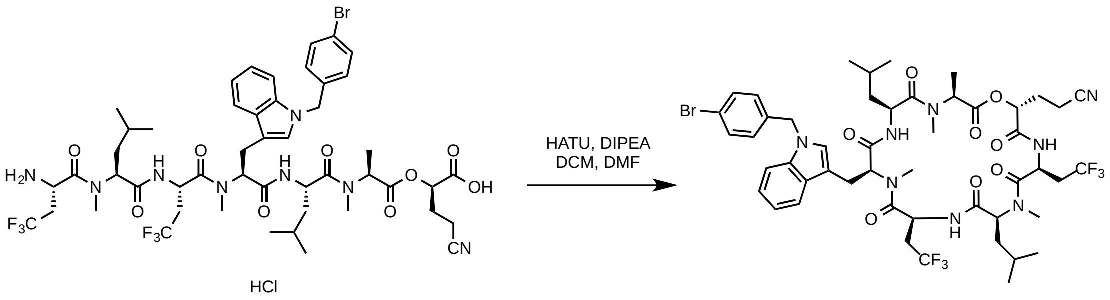

A solution of linear heptadepsipeptide (930 mg, 0.816 mmol) and DIPEA (427 µL, 2.45 mmol, 3 equiv.) in DCM (313 mL) was added, via a dropping funnel (at a rate of ~1 drop/sec), to a rapidly stirred solution of HATU (316 mg, 0.858 mmol), DMF (6 mL) and DCM (1.31 L). After complete addition, the funnel was rinsed into the reaction mixture with DCM (~10 mL) and the reaction was stirred for 18 h. An additional portion of HATU (158 mg, 429 mmol) was added and the reaction stirred for another 2 h. The reaction mixture was washed with HCl (500 mL of a 0.2 M aqueous solution), then with $NaHCO_3$ (500 mL of a 33% saturated aqueous solution). The DCM layer was then dried ($MgSO_4$), filtered and concentrated under reduced pressure. The residue purified by Flash chromatography (silica, step gradient elution, 5-50% acetone:hexane). Concentration of the appropriate fractions afforded **KZR-8445** (330 mg, 37%) as a white solid. **^{1}H-NMR** (400 MHz; acetone-d_6): δ 8.45 (d, *J* = 10.1 Hz, 1H), 8.25 (d, *J* = 9.6 Hz, 1H), 8.01 (d, *J* = 7.0 Hz, 1H), 7.71 (dd, *J* = 7.1, 0.9 Hz, 1H), 7.50-7.47 (m, 2H), 7.41 (t, *J* = 5.9 Hz, 1H), 7.36 (s, 1H), 7.18-7.09 (m, 4H), 5.45-5.34 (m, 2H), 5.26-5.06 (m, 3H), 4.76-4.71 (m, 1H), 4.37 (dd, *J* = 10.9, 3.6 Hz, 1H), 3.99-3.94 (m, 1H), 3.38-3.32 (m, 1H), 3.29 (s, 3H), 3.17-3.09 (m, 1H), 2.94 (s, 3H), 2.58 (s, 3H), 2.52-2.48 (m, 2H), 2.37-2.18 (m, 4H), 2.07 (dt, *J* = 4.4, 2.2 Hz, 2H), 1.96-1.82 (m, 2H), 1.57-1.45 (m, 8H), 1.04-0.94 (m, 10H), 0.90-0.85 (m, 2H), 0.49-0.42 (m, 1H). **LCMS** (ESI): Calculated for $C_{49}H_{62}BrF_6N_8O_8$ $[M+H]^+$, 1083.4; Found, 1083.2.

KZR-9508 was synthesized using the procedures described above for the synthesis of **KZR-8445**. **^{1}H NMR** (600 MHz, DMSO-d_6) δ 8.86 (d, *J* = 6.7 Hz, 1H), 8.33 (d, *J* = 10.1 Hz, 1H), 8.05 (d, *J* = 9.6 Hz, 1H), 7.54 (d, *J* = 7.9 Hz, 1H), 7.41 (d, *J* = 6.9 Hz, 1H), 7.17 (s, 1H), 7.13 (t, *J* = 7.5 Hz, 1H), 7.02 (t, *J* = 7.4 Hz, 1H), 5.33–4.87 (m, 3H), 4.47–3.96 (m, 4H), 3.36 (s, 1H), 3.20–3.12 (m, 3H), 3.08–2.97 (m, 1H), 2.86–2.78 (m, 3H), 2.60 (dd, *J* = 12.9, 8.4 Hz, 1H), 2.43 (s, 3H), 2.35 (ddd, *J* = 17.5, 8.4, 3.5 Hz, 1H), 2.21 (d, *J* = 2.1 Hz, 1H), 2.03–1.60 (m, 4H), 1.53–1.21 (m, 14H), 0.94–0.84 (m, 12H), 0.30–0.15 (m, 1H). **LCMS** (ESI): Calculated for $C_{44}H_{61}F_6N_8O_8+$ $[M + H]^+$, 943.4511; Found 943.4492.
