# Supplementary material for: Signal peptide mimicry primes Sec61 for client-selective inhibition
Source: Nat Chem Biol. 2023 May 11;19(9):1054–62. doi: 10.1038/s41589-023-01326-1 (PMC10449633; doi:10.1038/s41589-023-01326-1)
Supplement: Supplementary file 2 — Reporting Summary [file 41589_2023_1326_MOESM2_ESM.pdf]

nature research

Corresponding author(s): Ville O Paavilainen
Jack Taunton

Last updated by author(s): Feb 3, 2023

Reporting Summary

Nature Research wishes to improve the reproducibility of the work that we publish. This form provides structure for consistency and transparency in reporting. For further information on Nature Research policies, see our Editorial Policies and the Editorial Policy Checklist.

Statistics

For all statistical analyses, confirm that the following items are present in the figure legend, table legend, main text, or Methods section.

n/a	Confirmed	
☐	☒	The exact sample size (*n*) for each experimental group/condition, given as a discrete number and unit of measurement
☐	☒	A statement on whether measurements were taken from distinct samples or whether the same sample was measured repeatedly
☐	☒	The statistical test(s) used AND whether they are one- or two-sided<br>*Only common tests should be described solely by name; describe more complex techniques in the Methods section.*
☒	☐	A description of all covariates tested
☒	☐	A description of any assumptions or corrections, such as tests of normality and adjustment for multiple comparisons
☐	☒	A full description of the statistical parameters including central tendency (e.g. means) or other basic estimates (e.g. regression coefficient) AND variation (e.g. standard deviation) or associated estimates of uncertainty (e.g. confidence intervals)
☐	☒	For null hypothesis testing, the test statistic (e.g. *F*, *t*, *r*) with confidence intervals, effect sizes, degrees of freedom and *P* value noted<br>*Give P values as exact values whenever suitable.*
☒	☐	For Bayesian analysis, information on the choice of priors and Markov chain Monte Carlo settings
☒	☐	For hierarchical and complex designs, identification of the appropriate level for tests and full reporting of outcomes
☒	☐	Estimates of effect sizes (e.g. Cohen's *d*, Pearson's *r*), indicating how they were calculated

Our web collection on statistics for biologists contains articles on many of the points above.

Software and code

Policy information about availability of computer code

Data collection	Single particle cryo-EM data was collected on the eBIC Krios V in Oxford UK..<br><br>Gaussia luciferase and CellTiter-Glo data were collected on a Tecan infinite M1000 Pro plate reader. Cytokine data were collected on a Meso Scale Diagnostics MESO QuickPlex SQ 120 imager.<br><br>No software was used for data collection as clinical scores were scored for disease severity on a 0 (no disease) – 4 (maximal swelling) scoring system and summed for individual animal scores.<br><br>Simulations using the CHARMM force fields were performed using GROMACS simulation package v2021.3." "All analyses on the simulations performed using CHARMM force fields were performed using tools bundled in the GROMACS simulation package. Simulation snapshots were rendered using the tachyon renderer in VMD 1.9.4a51.
Data analysis	All data analysis software is commercially or publicly available and described in the Methods section.<br>IC50s were fit by Prism 8 (GraphPad).<br>Statistical analyses (two-way ANOVA followed by Bonferroni post hoc analysis) was performed using GraphPad Prism Software (version 7.01).<br>The following programs were used for single particle cryo-EM data analysis and structure visualization: Scipion v3, Relion v3.0 and v3.1, Motioncor2 v1.4.0, CTFfind4 v4.1.9, CrYOLO v1.7.6, UCF Chimera v1.16, UCSF ChimeraX C1.3, PHENIX V1.17, COOT v0.9.

For manuscripts utilizing custom algorithms or software that are central to the research but not yet described in published literature, software must be made available to editors and reviewers. We strongly encourage code deposition in a community repository (e.g. GitHub). See the Nature Research guidelines for submitting code & software for further information.

Data

Policy information about availability of data

All manuscripts must include a data availability statement. This statement should provide the following information, where applicable:

- Accession codes, unique identifiers, or web links for publicly available datasets
- A list of figures that have associated raw data
- A description of any restrictions on data availability

The data supporting the findings of this work are available within the article and its Supplementary Information files.
Coordinates and cryo-EM data and maps have been deposited in the PDB under accession code 7ZL3 and in the Electron Microscopy Data Bank under accession code EMD-14776. Micrograph movie files, motion corrected micrographs and particle files are deposited in EMPIAR under accession code EMPIAR-11405. All simulation inputs and outputs with the CHARMM force field are available at DOI: 10.5281/zenodo.6626602.

Field-specific reporting

Please select the one below that is the best fit for your research. If you are not sure, read the appropriate sections before making your selection.

☒ Life sciences ☐ Behavioural & social sciences ☐ Ecological, evolutionary & environmental sciences

For a reference copy of the document with all sections, see nature.com/documents/nr-reporting-summary-flat.pdf

Life sciences study design

All studies must disclose on these points even when the disclosure is negative.

Sample size	No statistical methods were used to pre-determine the sample size for cell culture experiments. Sample size (N ≥ 2) was chosen according to literature showing similar methods of analysis.<br>No statistical methods were used to pre-determine the sample size for the animal model experiments. Sample size (N ≥ 10) was chosen according to literature showing similar methods of analysis (Muchamuel et al. 2009, Nature Medicine PMID: 19525961).
Data exclusions	No data were excluded.
Replication	All cellular experiments were performed with at least three biological replicates.<br>Data for animal model experiments is representive of two separate experiments.
Randomization	Mice were randomized to treatment groups based on clinical scores. Randomization was not applicable to cell-based experiments as they did no experimental groups were involved.
Blinding	Individual animals from each experiment were analyzed in parallel using identical procedures and were not blinded. The clinical scores were determined by a standard procedure and therefore no blinding was performed.

Reporting for specific materials, systems and methods

We require information from authors about some types of materials, experimental systems and methods used in many studies. Here, indicate whether each material, system or method listed is relevant to your study. If you are not sure if a list item applies to your research, read the appropriate section before selecting a response.

Materials & experimental systems

n/a	Involved in the study	
☐	☒	Antibodies
☐	☒	Eukaryotic cell lines
☒	☐	Palaeontology and archaeology
☐	☒	Animals and other organisms
☒	☐	Human research participants
☒	☐	Clinical data
☒	☐	Dual use research of concern

Methods

n/a	Involved in the study	
☒	☐	ChIP-seq
☒	☐	Flow cytometry
☒	☐	MRI-based neuroimaging

Antibodies

Antibodies used	Polyclonal anti-actin antibody (Fisher Scientific #MS1295P1)<br>Polyclonal RPL18A antibody (Proteintech #14653-1-AP)<br>Sec61a Polyclonal antibody, (Novus Biologicals #NB120-15575), dilution 1:2000.<br>Strep-tag II antibody (BioRad #MCA2489).<br>β-Actin antibody Cell Signaling #(3700).

	IRDye ® 800CW Goat Anti-Mouse IgG Secondary Antibody (Li Cor #926-32210), dilution 1:10000.<br>Antibodies used in this study were obtained as part of Meso Scale Diagnostics U-PLEX multiplex kits: #K15067L for human, #K15069L for mouse.<br>Antibodies used in this study were obtained as part of Arthrogen-CIA® 5-Clone Cocktail Kit, 100 mg: #53100<br>Poly-clonal anti-SARS-CoV-NP antisera produced in a single rabbit, Garcia-Sastre lab at Mount Sinai.
Validation	The applications of the antibodies used in this study have been validated by the manufacturer based on their on-line statements, specifically:<br><br>For human cytokines, see: https://www.mesoscale.com/~/media/files/product%20inserts/u-plex%20biomarker%20group%201%20human%20insert-multiplex.pdf<br><br>For mouse cytokines, see: https://www.mesoscale.com/~/media/files/product%20inserts/u-plex%20biomarker%20group%201%20mouse%20multiplex%20insert.pdf<br><br>The applications of the antibodies used in this study have been validated by the manufacturer based on their on-line statements, specifically:<br><br>https://www.chondrex.com/products/arthrogen-cia-clone-cocktail-kit-mg<br><br>For Strep-tag II antibody, see https://www.bio-rad-antibodies.com/monoclonal/synthetic-peptide-strep-tag-classic-antibody-strep-tag-ii-mca2489.html?f=purified&_ga=2.82922901.2067593208.1654723376-569005095.1654723375<br><br>For β-Actin antibody, see https://www.cellsignal.com/products/primary-antibodies/b-actin-8h10d10-mouse-mab/3700<br><br>For IRDye 800CW goat anti-mouse IgG, see https://www.licor.com/bio/reagents/irdye-800cw-goat-anti-mouse-igg-secondary-antibody<br><br>For IRDye 800CW goat anti-rabbit IgG, see https://www.licor.com/bio/reagents/irdye-800cw-goat-anti-rabbit-igg-secondary-antibody

Eukaryotic cell lines

Policy information about cell lines

Cell line source(s)	HEK293T<br>Flp-In™ T-REx™ 293 Cell Line (ThermoFisher#R78007)<br>Vero E6 cells used at Mount Sinai and Institut<br>Pasteur were purchased from ATCC (VERO C1008 [Vero 76, clone E6, Vero E6] (ATCC® CRL-1586™)).
Authentication	Cell line was acquired from ThermoFisher and no additional authentication was performed.
Mycoplasma contamination	The cell lines were not tested for mycoplasma contamination.
Commonly misidentified lines (See ICLAC register)	No commonly misidentified cell lines were used.

Animals and other organisms

Policy information about studies involving animals; ARRIVE guidelines recommended for reporting animal research

Laboratory animals	8-12 weeks-old female BALB/c mice. Mice were housed as 5 mice per cage containing enrichment and were maintained on a 12 hour on/off light cycle. The relative ambient humidity was maintained at 55%+- 10%. The temperature was maintained between 68-78 F.
Wild animals	The study did not involve any wild animals.
Field-collected samples	The study did not involve samples collected from the field.
Ethics oversight	All in vivo studies were conducted in accordance with the current guidelines for animal welfare (National Research Council Guide for the Care and Use of Laboratory Animals, 2011). The procedures used were reviewed and approved by the Institutional Animal Care and Use Committee.

Note that full information on the approval of the study protocol must also be provided in the manuscript.
