# Supplementary figures and images for: Signal peptide mimicry primes Sec61 for client-selective inhibition
Source: Nat Chem Biol. 2023 May 11;19(9):1054–62. doi: 10.1038/s41589-023-01326-1 (PMC10449633; doi:10.1038/s41589-023-01326-1)

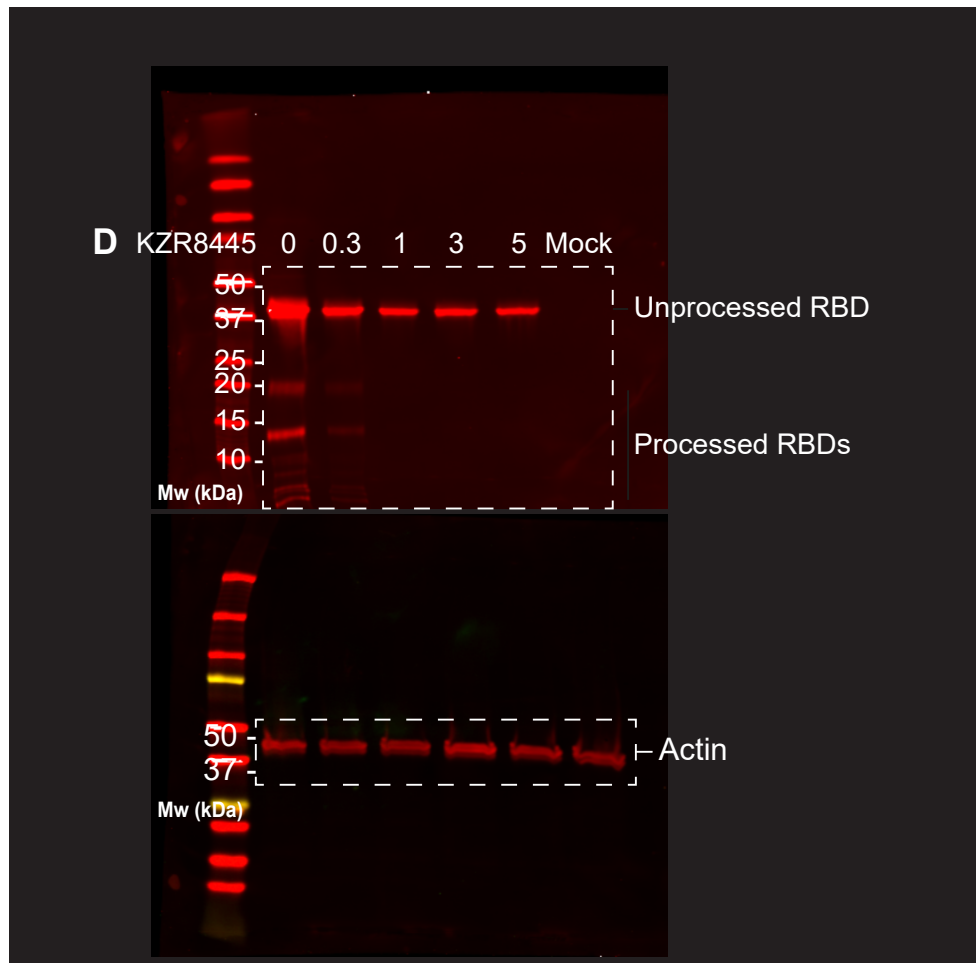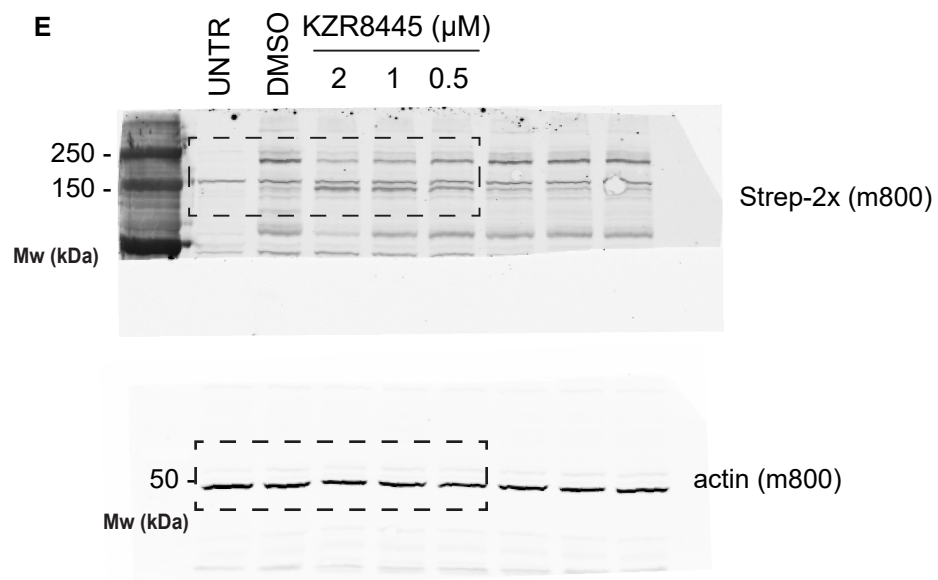

Supplement: Supplementary file 11 — Unprocessed western blots and gels. [file 41589_2023_1326_MOESM11_ESM.pdf]
