# Supplementary material for: Signal peptide mimicry primes Sec61 for client-selective inhibition
Source: Nat Chem Biol. 2023 May 11;19(9):1054–62. doi: 10.1038/s41589-023-01326-1 (PMC10449633; doi:10.1038/s41589-023-01326-1)
Supplement: Supplementary file 13 — Unprocessed western blots and gels. [file 41589_2023_1326_MOESM13_ESM.pdf]

A

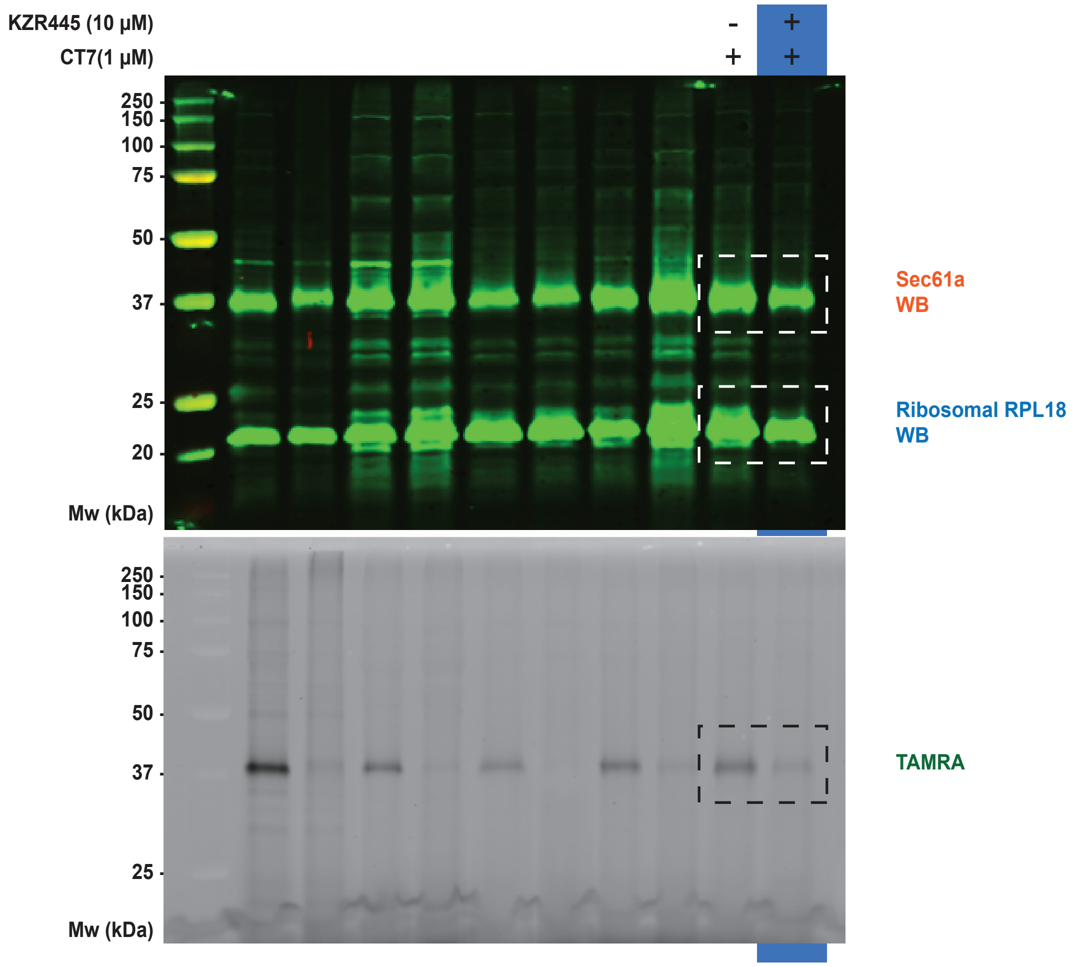
KZR445 (10 µM)
CT7(1 µM)
-
+
+
+
250
150
100
75
50
37
25
20
Mw (kDa)
Sec61a
WB
Ribosomal RPL18
WB
250
150
100
75
50
37
25
Mw (kDa)
TAMRA

C

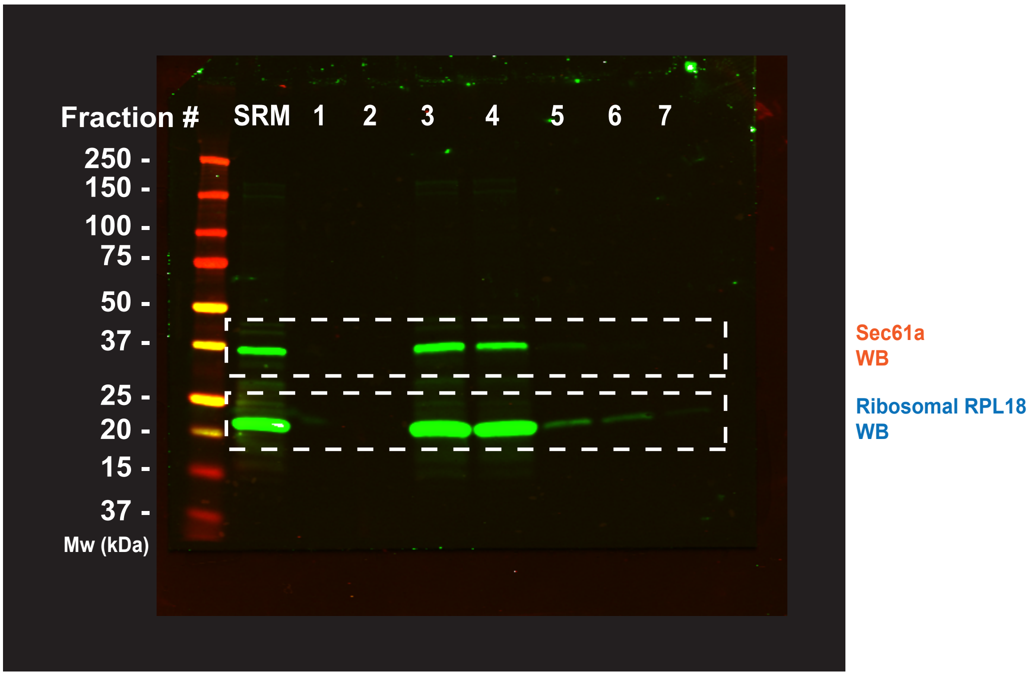
Fraction #
SRM
1
2
3
4
5
6
7
250
150
100
75
50
37
25
20
15
37
Mw (kDa)
Sec61a
WB
Ribosomal RPL18
WB
